# Supplementary material for: Economic evaluation of tranexamic acid for the treatment of acute gastrointestinal bleeding: a cost-effectiveness analysis using data from the HALT-IT randomised controlled trial
Source: BMJ Open. 2022 Jul 19;12(7):e060505. doi: 10.1136/bmjopen-2021-060505 (PMC9301815; doi:10.1136/bmjopen-2021-060505)
Supplement: Author's manuscript [file bmjopen-2021-060505.draft_revisions.pdf]

BMJ Open is committed to open peer review. As part of this commitment we make the peer review history of every article we publish publicly available.

When an article is published we post the peer reviewers' comments and the authors' responses online. We also post the versions of the paper that were used during peer review. These are the versions that the peer review comments apply to.

The versions of the paper that follow are the versions that were submitted during the peer review process. They are not the versions of record or the final published versions. They should not be cited or distributed as the published version of this manuscript.

BMJ Open is an open access journal and the full, final, typeset and author-corrected version of record of the manuscript is available on our site with no access controls, subscription charges or pay-per-view fees (http://bmjopen.bmj.com).

If you have any questions on BMJ Open's open peer review process please email info.bmjopen@bmj.com

BMJ Open

An economic evaluation of tranexamic acid for the treatment of acute gastrointestinal bleeding: A cost-effectiveness analysis using data from the HALT-IT randomised controlled trial

Journal:	*BMJ Open*
Manuscript ID	bmjopen-2021-060505
Article Type:	Original research
Date Submitted by the Author:	23-Dec-2021
Complete List of Authors:	Bazeer, Nuha; London School of Hygiene & Tropical Medicine, London, UK and London School of Economics and Political Science, London, UK<br>Miners, Alec; London School of Hygiene & Tropical Medicine, Department of Health Services Research and Policy<br>Roberts, Ian; London School of Hygiene & Tropical Medicine, Clinical Trials Unit<br>Shakur-Still, Haleema; London School of Hygiene & Tropical Medicine, Clinical Trials Unit<br>Jairath, Vipul; Western University, Department of Medicine, Division of Gastroenterology<br>Williams, Jack; London School of Hygiene & Tropical Medicine, Department of Health Services Research and Policy
Keywords:	GASTROENTEROLOGY, Bleeding disorders & coagulopathies < HAEMATOLOGY, HEALTH ECONOMICS

SCHOLARONE™
Manuscripts

BMJ

I, the Submitting Author has the right to grant and does grant on behalf of all authors of the Work (as defined in the below author licence), an exclusive licence and/or a non-exclusive licence for contributions from authors who are: i) UK Crown employees; ii) where BMJ has agreed a CC-BY licence shall apply, and/or iii) in accordance with the terms applicable for US Federal Government officers or employees acting as part of their official duties; on a worldwide, perpetual, irrevocable, royalty-free basis to BMJ Publishing Group Ltd ("BMJ") its licensees and where the relevant Journal is co-owned by BMJ to the co-owners of the Journal, to publish the Work in this journal and any other BMJ products and to exploit all rights, as set out in our licence.

The Submitting Author accepts and understands that any supply made under these terms is made by BMJ to the Submitting Author unless you are acting as an employee on behalf of your employer or a postgraduate student of an affiliated institution which is paying any applicable article publishing charge ("APC") for Open Access articles. Where the Submitting Author wishes to make the Work available on an Open Access basis (and intends to pay the relevant APC), the terms of reuse of such Open Access shall be governed by a Creative Commons licence – details of these licences and which Creative Commons licence will apply to this Work are set out in our licence referred to above.

Other than as permitted in any relevant BMJ Author's Self Archiving Policies, I confirm this Work has not been accepted for publication elsewhere, is not being considered for publication elsewhere and does not duplicate material already published. I confirm all authors consent to publication of this Work and authorise the granting of this licence.

For peer review only

An economic evaluation of tranexamic acid for the treatment of acute gastrointestinal bleeding: A cost-effectiveness analysis using data from the HALT-IT randomised controlled trial

Authors:

Nuha Bazeer[1] (0000-0002-4851-8402)

Alec Miners[2] (0000-0003-1850-1463)

Ian Roberts[3] (0000-0003-1596-6054)

Haleema Shakur-Still[3] (0000-0002-6511-109X)

Vipul Jairath[4] (0000-0002-1092-0033)

Jack Williams[2] (0000-0002-1331-387X)

1 London School of Hygiene & Tropical Medicine, London, UK and London School of Economics and Political Science, London, UK

2 Department of Health Services Research and Policy, London School of Hygiene & Tropical Medicine, London, UK

3 Clinical Trials Unit, London School of Hygiene & Tropical Medicine, London, UK

4 Department of Medicine, Division of Gastroenterology, Western University, London, Canada

For peer review only - http://bmjopen.bmj.com/site/about/guidelines.xhtml

Corresponding author:
Jack Williams
London School of Hygiene & Tropical Medicine
Room 142, 15-17 Tavistock Place, London, WC1H 9SH
Jack.Williams@lshtm.ac.uk

For peer review only

For peer review only - http://bmjopen.bmj.com/site/about/guidelines.xhtml

Abstract

Objective: To perform an economic evaluation of tranexamic acid (TXA) versus no TXA, in additional to current clinical practice, for acute gastrointestinal bleeding, using the results of the HALT-IT trial, a large randomised controlled trial of 12,009 patients.

Design: A cost-effectiveness modelling analysis, performed over a lifetime time horizon.

Setting: The analysis was performed from a UK health service perspective

Participants: The model includes adults with acute gastrointestinal bleeding.

Outcomes measures: The model reports costs in Great British Pounds (GBP) in 2018 and outcomes as life years (LYs) and quality-adjusted life years (QALYs). Cost-effectiveness was evaluated using incremental cost-effectiveness ratios (ICERs), reported as the cost per QALY gained.

Methods: A Markov model was developed to calculate the overall costs and health outcomes of TXA administration versus no TXA. The model used data of the treatment effectiveness from the HALT-IT trial, which showed that TXA administration for acute gastrointestinal bleeding did not reduce all-cause mortality (RR 1.03, 95% CI 0.92; 1.16) compared to no TXA. Data on health-related quality of life, costs, and long-term mortality risks were derived from the literature. Costs and effects are discounted at 3.5% per annum.

Results: TXA was associated with marginally fewer LYs and QALYs, and lower costs, than treatment without TXA. The ICER associated with no TXA was £1,413 per LY gained and £1,981 per QALY gained. No TXA was 66% likely to be cost-effective at a £20,000 willingness to pay threshold, whilst TXA was 34% likely to be cost-effective.

Conclusion: Though inexpensive, TXA administration for patients with acute gastrointestinal bleeding is unlikely to be cost-effective.

For peer review only - http://bmjopen.bmj.com/site/about/guidelines.xhtml

Strengths and limitations of this study

- This is the first economic evaluation of tranexamic acid for the treatment of acute gastrointestinal bleeding
- Data for the treatment effect comes from the HALT-IT trial, which randomised 12,009 patients to either tranexamic acid or placebo.
- The cost-effectiveness model extrapolates the impact of tranexamic acid treatment over a lifetime time horizon, as recommended by the National Institute for Health and Care Excellence, for UK based economic evaluations
- The HALT-IT trial data only reports outcomes up to 28 days following the onset of acute gastrointestinal bleeding, and therefore the model only accounts for treatment effects observed within this 28 day trial period.

For peer review only

Introduction

Acute gastrointestinal bleeding (AGIB) refers to bleeding arising from the upper or lower gastrointestinal tract. Acute upper gastrointestinal bleeding (AUGIB) occurs more commonly than acute lower gastrointestinal bleeding (ALGIB) and has a higher case fatality rate of up to 10% [1]. AUGIB is a common medical emergency and results in approximately 85,000 cases each year within the United Kingdom, with around 4,000 AUGIB related deaths annually [2]. Thus, AUGIB presents a significant health concern with an estimated incidence of 133 cases per 100,000 population, accounting for 8% of all acute hospital admissions [2]. The leading causes of AUGIB have been recorded as peptic ulcers and esophagogastric varices [1, 3]. While peptic ulcers are often associated with long-term use of non-steroid anti-inflammatory drugs (NSAIDs), esophagogastric varices are typically complications of liver cirrhosis [4]. Comparisons of national AUGIB audits in the United Kingdom carried out in 1994 and 2007 have shown improved outcomes amongst patients, likely through advancements in endoscopic and some pharmacological therapies [1]. However, over the past two decades, there have been no significant changes to mortality associated with AUGIB, and mortality amongst older patients with other comorbidities remains high at 10% [2, 5]. Within the UK, annual in-hospital costs for all AUGIB cases have been estimated as £155.5 million, of which £12.6 million accounts for blood product transfusions (BPT) alone [6]. As the second most common medical reason for transfusion, GI bleeding accounts for 14% of all BPT within the UK, with inappropriate use recorded in 20% of cases [7, 8].

Tranexamic acid (TXA) is an anti-fibrinolytic agent which reduces fibrinolysis and inhibits the breakdown of blood clots [9]. It's used to treat women with heavy menstrual bleeding (menorrhagia) and people with haemophilia during dental extractions [10-13]. The efficacy of TXA has also been demonstrated in reducing blood loss during surgical operation trials [9]. It is widely available, relatively inexpensive, and can be administered through oral or intravenous formulations, without specialist monitoring [14]. Large, multi-centre, randomised controlled trials (RCTs) such as the WOMAN and CRASH-2 and -3 trials have shown TXA to significantly reduce bleeding mortality amongst patients at risk of postpartum haemorrhage and bleeding trauma patients respectively [15-17]. However, the timing of TXA administration is of particular importance, with no evidence of a benefit upon mortality when administered more than three hours after injury in both WOMAN and CRASH-2 and -3 trials. [16, 18]. Unlike postpartum haemorrhage or brain injury however, the onset of a gastrointestinal bleed may be less discernible, with presentation often delayed due to symptoms only becoming

For peer review only - http://bmjopen.bmj.com/site/about/guidelines.xhtml

visible when most severe. This was observed within the HALT-IT trial, with over 80% of patients presenting more than three hours after the onset of bleeding [19].

Previously conducted smaller RCTs in the UK have found no evidence of a difference in mortality, rebleeding, and the requirement of surgery [20, 21]. However, a 2014 Cochrane systematic review and meta-analysis of RCTs comparing TXA with placebo for AUGIB estimated large reductions in all-cause mortality, with a pooled risk ratio (RR) of 0.6 (95% CI, 0.42–0.87) across 8 studies with 1701 participants [22]. However, the mortality benefit of TXA was uncertain when excluding trials with a high risk of bias. For this reason, the authors concluded that tranexamic acid cannot be recommended for routine clinical practice. An updated systematic review and meta-analysis found similar findings, with a risk ratio of 0.59 (95% CI 0.43-0.82), and also concluded that high-quality RCTs were needed [23].

TXA is not currently recommended within UK guidelines for AUGIB or ALGIB [24]. National Institute for Health and Care Excellence (NICE) guidelines for AUGIB refer to limited evidence of an effect of TXA on mortality, but do not include recommendations for TXA in the absence of UK marketing authorisation for this indication. Similarly, guidelines from the British Society of Gastroenterology state that the mortality benefit of TXA is uncertain when excluding trials with a low risk of bias, suggesting recommendations are made following the HALT-IT trial [25].

The HALT-IT trial was a large multinational RCT that compared TXA versus placebo, in addition to current practice, in 12,009 people with AGIB [19]. The trial did not find evidence that TXA significantly reduces any of the key outcome measures, such as death due to bleeding within 5 days of randomisation (RR 0.99; 95% CI 0.82; 1.18) or all-cause mortality (RR 1.03 95% CI 0.92; 1.16). This means by default, even if relatively inexpensive, TXA is unlikely to represent value for money in this indication. However, there is still a need to perform an economic evaluation based on the HALT-IT results, in order to establish the level of uncertainty about this conclusion and to assess the extent to which only including the results from the systematic review would have impacted the cost-effectiveness estimates. The full results of the HALT-IT trial, including the economic results, are available in a separate National Institute of Health Research report [26].

For peer review only

Methods

Analysis

The base case economic analysis assessed the cost-effectiveness of treating patients identified as sustaining an AGIB with TXA versus no TXA, in addition to current practice, using estimates of treatment effect from the HALT-IT trial. A cost-effectiveness model was developed in Microsoft Excel and was analysed over a lifetime time horizon, from a UK NHS health service perspective, in line with UK NICE guidelines [27]. All costs are reported in 2018 Great British Pounds. Health outcomes were assessed as life years (LYs), and quality-adjusted life years (QALYs). All future costs and health outcomes were discounted at 3.5% per annum, as per NICE guidance [27]. We report the results as incremental cost-effectiveness ratios (ICERs) and incremental net monetary benefits (INMBs). A willingness to pay threshold of £20,000 per QALY has been used to determine cost-effectiveness, in line with NICEs Technology Appraisals recommendations [27].

The HALT-IT trial included a number of sub-group analyses, but as no treatment effects were observed amongst subgroups, they were not considered in the model. Whilst the trial consisted mostly of patients with AUGIB (89%), the model population includes both AUGIB and ALGIB, as per the overall trial population.

Model Structure

An existing Markov model of TXA following traumatic brain injury was adapted to estimate the cost-effectiveness of TXA for patients with AGIB [28]. The model consists of two health states *alive* and *dead*; as shown in Figure 1. People with AGIB enter the model in the *alive* health state. They progress to the *dead* health state over the initial 28-day period (the HALT-IT trial period) using daily data taken directly from the trial on all-cause deaths from the placebo arm of HALT-IT; this includes data for all UK and non-UK participants. For the remaining 337 days of the first year, the daily probability of death in the no-TXA arm was estimated using UK general population age-related mortality statistics [29], adjusted using a standardised mortality ratio (SMR) to reflect the long-term risk of excess death associated with surviving an AGIB [30]. In the base case, this SMR is applied for the entire time horizon. After this initial year period, the model uses an annual cycle length, and applies a probability of death based on UK all-cause mortality data, combined with the SMR. In line with the HALT-IT trial, the mean age of people entering the model was assumed to be 58.1 years (Table 1).

For peer review only - http://bmjopen.bmj.com/site/about/guidelines.xhtml

The SMR associated with the risk of death following an AGIB compared to that of the general population was derived from a study by Crooks et al (2013) [30]. It linked longitudinal data from the UKs Hospital Episodes Statistics dataset, General Practice Research Database and Office of National Statistics death register for people with non-variceal bleeds between 1997 and 2010. Controls were matched by age, sex, practice, and year. The study reported the risk of death was highest in the first year following an AGIB (SMR 5.21 95% CI 4.25; 6.38), and remained elevated from year 2 onwards (SMR 1.74 95% CI 1.42; 2.13). In the deterministic sensitivity analysis, excess mortality was excluded after 1 year.

Treatment Effect

The HALT-IT trial results for the primary outcome measure, death due do bleeding within 5 days from randomisation, was similar in both treatment arms (RR 0.99; 95% CI 0.82; 1.18), although the mean estimate slightly favoured TXA.[19] However, as the risks of venous thromboembolic events (RR 1.85; 95% CI 1.15; 2.98), death from malignancy (RR 1.63; 95% CI 1.10; 2.42), and seizure (RR 1.73; 95% CI 1.03; 2.93) were higher in the TXA- than placebo-group, the base case RR of all-cause mortality was used to estimate the overall effect of TXA treatment on survival (RR 1.03 95% CI 0.92; 1.16), and is shown in Table 1. This RR is based on all trial participants and is not UK specific since the subgroup analysis did not reveal any differences in treatment effect by country income level.

Utilities

Utility data were not collected as part of HALT-IT, therefore values were sourced from the existing literature. Only one directly relevant study could be identified – the relatively recent TRIGGER RCT [31]. The EQ-5D-3L questionnaire was administered at discharge or day-28, with an estimated utility of 0.735 (95% CI 0.70; 0.77) for those surviving an AGIB (Table 1) [6].

In line with previous economic evaluations of TXA, this single utility value of 0.735 was applied to all people who were alive at day-28, both during the initial 28-day period and at all times thereafter until death, in both treatment arms [28]. Note that functional status was measured in HALT-IT using the Katz Index of Independence in Activities of Daily Living [32], with the scores similar in both arms (score of 5.5 for both). An age adjustment was also applied to the utility value each cycle using the decrements reported by Kind et al., so that overall utility declined with age [33]. In a sensitivity analysis, the utility value of 0.735 after 1 year was

For peer review only - http://bmjopen.bmj.com/site/about/guidelines.xhtml

replaced with age-adjusted population values also reported by Kind et al, whereby the utility of those after the first year increased to be equal to the UK general population utility values. The latter approach mimics survivors of AGIB returning to 'full population health' after 1 year, however given the high prevalence of comorbidities in this population it is considered an optimistic scenario.

Costs

Treatment costs

The resource use associated with providing TXA treatment were derived from the HALT-IT trial and by making a number of assumptions. The total TXA dose (4g) included a loading- and maintenance-dose, for which the total cost was derived from the British National Formulary (£12 per patient) [34]. The cost of equipment for treatment was assumed to include a needle (£0.04), syringe (£0.06), sodium chloride infusion bag (£0.55) and isotonic IV solution (£3.77). To account for the administration time of TXA, hourly staff costs were taken from the Personal Social Services Research Unit for 2018 [35]. The model assumed that a Band 5 nurse would take 21 minutes to administer TXA (£12.95), following the treatment administration and expert guidance used within the economic evaluations of the CRASH [28, 36] and WOMAN trials [37]. This led to a total TXA treatment and administration cost of £29.37 per person (Table 2).

Inpatient stay costs

Information from HALT-IT was used to derive the costs of inpatient stays during the initial 28 day trial period. The total mean lengths of stay recorded by people receiving TXA and placebo were 5.83 and 5.80 days respectively (Table 2). In both treatment arms, 0.4 of these days were spent in intensive care units (ICU). These lengths of stay were multiplied by NHS Reference Costs for 2017/18 for non-elective stays to derive mean hospital costs for TXA and no TXA of £2,292 and £2,284 per person respectively [38].

Procedures

While in hospital during the 28-day trial period, participants underwent a number of procedures, including endoscopy, surgery and radiological interventions (Table 2). The cost of these procedures were calculated for each trial arm using the probability of occurrence from the HALT-IT trial multiplied by the NHS Reference Costs for 2017/18. The mean per person procedural costs for the TXA and no TXA treatment options were £2,216 and £2,260 respectively.

For peer review only - http://bmjopen.bmj.com/site/about/guidelines.xhtml

Transfusions

While in hospital, people received transfusions of blood / red blood cells, fresh frozen plasma and platelets (Table 2). Information from HALT-IT on the frequency and the mean number of units for each transfusion type were combined with unit costs from the NHS Blood and Transfusions price list to generate overall costs [39]. The mean per person transfusion costs for the TXA and no TXA treatment options were £234 and £245 respectively.

Post discharge costs

The HALT-IT and TRIGGER trials observed participants for a maximum of 28-days, therefore longer-term care costs were sourced from the literature. A specific study related to AGIB could not be identified. However, a recent UK study by Ramagopalan et al. quantified the costs of gastrointestinal bleeding among people with nonvalvular atrial fibrillation (NVAF), who were and were not experiencing a bleed, and used a difference in difference approach to calculate the costs attributable to a gastrointestinal bleed (cases, n=7,753 and controls, n=7,753) [40].

The study used 2017/18 NHS reference costs and calculated the annual costs of caring for people with a bleed to be £3,989, £1,816 and £1,777 in the first second- and third-years post-bleed (Table 2). In the absence of other information, the reported year three costs were inputted into the model for years four onwards, although they were assumed to be one third the original amount. The standard errors that the costing study reports could not be easily incorporated into our analysis (the statistical approach used allowed costs to be negative), therefore they were assumed to be 50% of the mean value in all instances, which is thought to reflect a relatively large degree of uncertainty around the mean estimates. The importance of these assumptions was assessed in the sensitivity analysis.

Sensitivity Analysis

Various one-way deterministic sensitivity analyses were undertaken to assess the robustness of the results to alternative assumptions. In line with NICE guidelines, one-way DSA was conducted on the discount rate for both costs and health outcomes, using values of 0% and 6%. It was also conducted on utility inputs of the model, using the higher and lower values for health-related quality of life for AGIB survivors.

For peer review only - http://bmjopen.bmj.com/site/about/guidelines.xhtml

To simultaneously investigate the effect of parameter uncertainty, probabilistic sensitivity analysis (PSA) was also undertaken using 10,000 Monte Carlo simulations; the parameter distributions are specified in Table 1 and Table 2. The probabilistic results are presented on a cost-effectiveness plane, and as a cost-effectiveness acceptability curve (CEAC). Parameters that were excluded from the PSA are not assigned a distribution type in the tables.

A scenario analysis was performed to consider the economic results that would have been generated prior to the availability of the results of TXA from the HALT-IT trial. This analysis was performed primarily to demonstrate the differences in decision making when using low or moderate quality evidence to populate the economic model, particularly from trials with a high risk of bias. In this analysis the 28-day mortality risk was 0.084 (71/850) in the control group, whilst the TXA all-cause mortality RR was 0.60 (95% CI: 0.42-0.87), as per the Cochrane analysis [22].

Patient and Public Involvement

Patient and public input was used to inform the HALT-IT trial procedures, including study information sheets and consent forms.[26] Patients stated that the research should account for quality of life issues in additional to the treatment efficacy and safety, which this economic analysis does. There was no patient or public involvement specifically in the design of the economic model.

For peer review only - http://bmjopen.bmj.com/site/about/guidelines.xhtml

Results

Base-Case Analysis

The base case results are shown in Table 3. In both the deterministic and probabilistic analyses TXA was associated with marginally fewer LYs and QALYs, and marginally lower costs, than treatment without TXA. The deterministic QALYs and costs associated with TXA and no-TXA were 8.58 and 8.61, and £16,706 and £16,764 respectively. The associated ICERs are £1,413 per LY gained and £1,981 per QALY gained if no-TXA is used instead of TXA, meaning no TXA is preferred at the NICE willingness to pay threshold, or alternatively that providing TXA is not cost-effective. The deterministic and mean probabilistic results are very similar (Table 3).

The lower mean health outcomes for TXA are driven by the fact the mean risk ratio for all-cause mortality (i.e. the relative treatment effect) slightly favours the no-TXA treatment arm (RR 1.03). The higher incremental costs associated with no-TXA administration are largely driven by the fact people who did not receive TXA live for marginally longer than those who received TXA, thus they incur slightly higher post discharge costs, and marginally higher costs for procedures and transfusions, exceeding the additional low cost of TXA administration.

Probabilistic Sensitivity Analysis

The cost-effectiveness plane shows that the PSA simulations are clustered around the origin and distributed relatively evenly across all four cost-effectiveness quadrants (Figure 2). This is because the point estimate for the risk ratio of all-cause mortality is only slightly above one, and is included within its confidence interval (RR 1.03 95% CI 0.92; 1.16). Therefore, there is little to choose between the two treatment options in terms of outcomes. The same is also true of the relatively low cost of TXA administration (£29.37 per person), meaning that the majority of the costs incurred are the hospital costs (hospital stay, procedures and transfusions) and post-discharge costs.

The CEAC (Figure 3) shows that at any willingness to pay for an additional QALY above £2,000, no-TXA is likely to be the more cost-effective option. At the NICE specified willingness to pay threshold levels of £20,000 and £30,000 per additional QALY, TXA has a 34% and 33% probability of being the most cost-effective option, respectively. Note that the base case CEAC does not indicate that there is a 0% probability TXA is the most cost-effective treatment option, irrespective of the willingness to pay value. TXA treatment produced more QALYs at lower cost (i.e. it was the 'dominant' treatment option) in 15% of simulations. Rerunning the PSA

For peer review only - http://bmjopen.bmj.com/site/about/guidelines.xhtml

For peer review only

assuming the post discharge costs had a standard error of 20%, instead of 50%, of their mean value, had a negligible impact on the probability of TXA being cost-effective.

Deterministic Sensitivity Analysis

Various deterministic one-way sensitivity analyses are shown Table 4. In all but one instance the results show that the base case ICER was very robust to alternative model parameter and structural assumptions. The exception is when the risk ratio of all-cause mortality is reduced to 0.92 (the lower bound of the 95% CI). In this instance, TXA produces marginally better health outcomes at slightly higher costs than no-TXA, producing an ICER of £1,144 per QALY gained if TXA is used instead of no TXA. Note that even when the cost of TXA and its administration was set to £0 instead of the base case value of £29.37, the ICER per QALY gained remained under £3,000, favouring no-TXA.

Meta-analysis scenario analysis

When parameterising the model with the mortality risk and all-cause mortality treatment effect reported from a meta-analysis containing multiple studies at risk of bias, prior to the availability of the HALT-IT trial, TXA becomes highly cost-effective, with an ICER of £955 per LY gained and £1,337 per QALY gained. In the probabilistic analysis, TXA was 99.5% likely to be cost-effective at the £20,000 per QALY threshold.

For peer review only - http://bmjopen.bmj.com/site/about/guidelines.xhtml

Discussion

The results from the base case analysis suggest that the costs and outcomes of treating people with AGIB with and without TXA, as enrolled and treated in the HALT-IT trial, are very similar. However, the base case probabilistic analysis showed that there is a 34% chance TXA is the most cost-effective option at £20,000 per QALY thresholds. Despite being a relatively inexpensive intervention, TXA is unlikely to be a cost-effective use of NHS resources [19], and no-TXA is likely to be the most cost-effective option. These results are in line with our prior expectations given the additional, albeit modest, cost of providing TXA, which did not reduce mortality.

As with all decision models, our analysis includes a number of parameter and structural assumptions. However, extensive sensitivity analysis showed that they were generally unimportant in terms of changing the conclusion that TXA is unlikely to be cost-effective in this indication. The only sensitivity analysis that changed this conclusion was when TXA reduced mortality. Given that TXA resulted in marginally lower costs than no TXA, it would be cost-effective if it resulted in any all-cause mortality benefit. Given this, if future research identified a modest treatment effect for a subgroup of AGIB patients, then TXA would likely be cost-effective in this group.

To the best of our knowledge, no other cost-effectiveness analyses of TXA for people with AGIB have been reported. However, economic evaluations based on the series of CRASH RCTs and WOMAN RCT have concluded TXA is highly cost-effective following trauma injury and post-partum haemorrhage [15, 16, 28, 37]. The dose of TXA used in the HALT-IT trial is higher than that used in the previous studies, but this increased cost is negligible. The reason for these differences in the cost-effectiveness results is driven by the mortality benefit associated with TXA in other indications, which was not observed the HALT-IT trial population, or in any sub-group analyses. Of considerable importance and highlighted within the WOMAN and CRASH-3 studies was the time to treatment, with earlier treatment (within 3 hours of onset of bleeding) associated with improved outcomes [41]. In AGIB patients, it is often difficult to identify the onset of bleeding, and presentation is often delayed. In the HALT-IT trial, 80% of participants received treatment more than 3 hours after the suspected onset of bleeding. Furthermore, patients with AGIB are older, with more comorbidities [19].

One of the main strengths of this analysis is that it is the first economic evaluation of TXA for people with AGIB based on the results of a large RCT. The results from HALT-IT are in contrast to previous meta-analyses of small trials, in which TXA was estimated to result in a large reduction in all-cause mortality in people with AUGIB, when including trials at risk of bias [22, 23]. A scenario analysis using these meta-analysis results

For peer review only - http://bmjopen.bmj.com/site/about/guidelines.xhtml

found that, had an economic evaluation using the results of this meta-analysis been performed prior to the HALT-IT trial, the results would have suggested TXA was almost certainly cost-effective, with little uncertainty, and indicated little value in performing additional research. With hindsight, we believe the authors of these analyses were correct to conclude that their findings were insufficient to recommend the routine use of TXA in clinical practice, since this would have potentially led to poorer outcomes at a health systems level and potentially impacted on HALT-IT recruitment. For future health economic analyses, we suggest that researchers should be cautious in their use of meta-analyses that include trials considered susceptible to error or bias, which could subsequently result in misleading policy recommendations.

In carrying out an economic evaluation with robust sensitivity analysis, our findings emphasise the importance of large RCTs designed to ascertain the effectiveness of an intervention, and the importance of a thorough economic analysis using high quality data, to assess the cost-effectiveness of low-cost interventions.

Conclusion

Building on the evidence provided from the largest RCT in patients with AGIB, the results of this analysis have shown TXA administration for patients with AGIB is, on balance, unlikely to be cost-effective at UK WTP thresholds. However, there remains uncertainty around this conclusion, with TXA being the most cost-effective treatment option in approximately one-third of simulations. This is because TXA would be cost-effective if it resulted in any mortality benefit, and the confidence interval for the all-cause mortality treatment effect observed in the HALT-IT trial (0.92-1.16), does not rule out such a finding. These results also highlight that if a modest treatment effect for a subset of patients can be identified in the future, then TXA has considerable scope to be cost-effective for this group.

For peer review only - http://bmjopen.bmj.com/site/about/guidelines.xhtml

Tables

Table 1: Base case model input parameters

Parameter	Value	95% CIs	Distribution	Source
Age at model start	*58.1*	-	-	HALT-IT
SMR year 1	*5.21*	4.25; 6.38	Log normal	Crooks et al [30]
SMR year 2	*1.74*	1.42; 2.13	Log normal	Crooks et al [30]
RR of all-cause mortality	*1.03*	0.92; 1.16	Log normal	HALT-IT
28-day probability of death^	*0.092*	0.084; 0.099	Beta	HALT-IT
Utility AGIB survivor	*0.735*	0.70; 0.77	Beta	Campbell et al [6]
Utility decrements by age (years)				Kind et al [33]
55-64	*0*	-	-	
65-74	*0.02*	-	-	
≥75	*0.07*	-	-	
Utility UK general population	*By age*	-	-	Kind et al [33]
All-cause mortality	*By age*	-	-	ONS [29]
Discount rate costs	*3.5%*	-	-	NICE [27]
Discount rate QALYs	*3.5%*	-	-	NICE **[27]**

^The 28-day risk of death in the placebo arm was sampled from a beta distribution during the probabilistic sensitivity analysis, based on the proportion of deaths during the trial period (548/5981). The deaths occurring on each day, as a proportion of the overall 28-day risk, remained the same during sampling.

For peer review only

1
2 *Table 2: Base case cost parameters*

Parameter	Value	SE or proportion	Distribution	Source
TXA administration	*£29.37*	-	-	BNF [34], Assumption
Inpatient stays to day-28				
Total length of stay (days)				
TXA	*5.83*	1.46	Gamma	HALT-IT
No TXA	*5.80*	1.47	Gamma	HALT-IT
Days in ICU				
TXA	*0.4*	0.46	Gamma	HALT-IT
No TXA	*0.4*	0.51	Gamma	HALT-IT
Unit cost per day non-ICU	*£313*	-	-	Reference costs [38]
Unit cost per day on ICU	*£1,479*	-	-	Reference costs [38]
Procedures to day-28				
Prob endoscopy - diagnostic				
TXA	*0.8*	4,781/5,953	Beta	HALT-IT
No TXA	*0.79*	4,729/5,978	Beta	HALT-IT
Unit cost	*£617*	-	-	Reference costs [38]
Prob endoscopy - therapeutic				
TXA	*0.43*	2,542/5,953	Beta	HALT-IT
No TXA	*0.44*	2,658/5,978	Beta	HALT-IT
Unit cost	*£721*	-	-	Reference costs [38]
Prob surgical intervention				
TXA	*0.02*	146/5,953	Beta	HALT-IT
No TXA	*0.03*	158/5,978	Beta	HALT-IT
Unit cost	*£1,065*	-	-	Reference costs [38]
Prob radiological - diagnostic				
TXA	*0.29*	1,704/5,953	Beta	HALT-IT
No TXA	*0.29*	1,744/5,978	Beta	HALT-IT
Unit cost	*£4,625*	-	-	Reference costs [38]
Prob radiological - therapeutic				
TXA	*0.01*	74/5,953	Beta	HALT-IT
No TXA	*0.01*	89/5,978	Beta	HALT-IT
Unit cost	*£4,625*	-	-	Reference costs [38]
Transfusions				
Prob blood or red cells				
TXA	*0.67*	3,984/5,951	Beta	HALT-IT
No TXA	*0.67*	4,018/5,978	Beta	HALT-IT
Mean units of blood or red cells				
TXA	*2.80*	0.61	Gamma	HALT-IT
No TXA	*2.90*	0.69	Gamma	HALT-IT
Unit cost	*£122*	-	-	NHS Blood & trans. [39]
Prob fresh frozen plasma				
TXA	*0.15*	910/5,951	Beta	HALT-IT
No TXA	*0.16*	993/5,978	Beta	HALT-IT
Mean units fresh frozen plasma				
TXA	*0.90*	0.61	Gamma	HALT-IT
No TXA	*1.0*	0.66	Gamma	HALT-IT
Unit cost	*£28*	-	-	NHS Blood & trans. [39]
Prob platelets				
TXA	*0.04*	219/5,951	Beta	HALT-IT
No TXA	*0.04*	255/5,978	Beta	HALT-IT
Mean units of platelets				
TXA	*0.20*	0.23	Gamma	HALT-IT
No TXA	*0.20*	0.26	Gamma	HALT-IT
Unit cost	*£178*	-	-	NHS Blood & trans. [39]
Post discharge costs				
Year1	*£3,989*	£1,995*	Gamma	Ramagopalan et al. [40]
Year 2	*£1,816*	£908*	Gamma	Ramagopalan et al. [40]
Year 3	*£1,777*	£889*	Gamma	Ramagopalan et al. **[40]**

3 *standard errors were assumed to be 50% of the mean value in the base case analysis
4

For peer review only - http://bmjopen.bmj.com/site/about/guidelines.xhtml

1 *Table 3: Base case probabilistic results*

	Costs (£)	LYs	QALYs	ICER LYs (£)	ICER QALYs (£)
Deterministic					
No TXA	*16,764*	12.08	8.61	1,413	1,981
TXA	*16,706*	12.04	8.58	-	-
Probabilistic (PSA)					
No TXA	*16,895*	12.21	8.70	1,419	1,988
TXA	*16,840*	12.17	8.67	-	-

2

For peer review only

For peer review only - http://bmjopen.bmj.com/site/about/guidelines.xhtml

Table 4: Deterministic one-way sensitivity analysis results

	Inc. costs (£)	Inc. LY	Inc. QALY	ICER LY (£)	ICER QALY (£)	INMB (QALY) (£)
**Base case**	-58	-0.04	-0.03	1,413	1,981	-524
Excess mortality excluded after first year	-62	-0.05	-0.03	1,306	1,843	-611
Monitoring costs (full monitoring costs for year 4 onwards)	-96	-0.04	-0.03	2,357	3,304	-486
Monitoring costs not included beyond 3 years	-38	-0.04	-0.03	941	1,319	-544
0% discount rate	-70	-0.06	-0.04	1,162	1,646	-783
6% discount rate	-52	-0.03	-0.02	1,609	2,243	-412
Utility - general population utility after first year	-58	-0.04	-0.03	1,413	1,826	-574
Monitoring costs - inpatient costs excluded in year 1	-49	-0.04	-0.03	1,204	1,688	-533
TXA all-cause mortality risk ratio (0.92)	79	0.10	0.07	816*	1,144*	1,310
TXA all-cause mortality risk ratio (1.16)	-210	-0.19	-0.14	1,080	1,514	-2,568
Total TXA administration at £0	-87	-0.04	-0.03	2,133	2,990	-600

*In this scenario, the ICERs flip meaning they favour TXA treatment (hence the positive incremental net monetary benefits [INMBs]); INMBs calculated using a £20,000 willingness to pay for an additional LY / QALY threshold; negative costs, LYs and QALYs indicate that TXA is less costly and less effective than no TXA

review only

For peer review only - http://bmjopen.bmj.com/site/about/guidelines.xhtml

Figures

Figure 1: Markov Model structure showing both health states within the model.

For peer review only

For peer review only - http://bmjopen.bmj.com/site/about/guidelines.xhtml

Figure 2: Base case cost-effectiveness plane

The NE quadrant contains 15% of simulations; SW quadrant 37%; SE quadrant 14%; NW quadrant 34%

For peer review only

1 *Figure 3: Base case cost-effectiveness acceptability curve*

2

3

For peer review only

For peer review only - http://bmjopen.bmj.com/site/about/guidelines.xhtml

Statements

Contributorship statement: All authors contributed to the study conception and design. The economic model was developed by NB and JW. The economic analyses were performed by NB, AM, and JW. The first draft of the manuscript was written by NB, and all authors commented on previous versions of the manuscript. All authors read and approved the final manuscript.

Competing interests: The authors declare that they have no financial or non-financial interests to disclose, and no conflicts of interest to declare.

Funding: This work was supported by the UK National Institute for Health Research Health Technology Assessment Programme (HTA/11/01/04).

Data sharing statement: For the HALT-IT trial data, individual de-identified patient data, including data dictionary, will be made available via our data sharing portal, The Free Bank of Injury and Emergency Research Data (freeBIRD) website indefinitely (https://freebird.lshtm.ac.uk/). The economic model is available from the authors upon request.

Ethics approval: The HALT-IT trial was approved by the UK NRES Committee East of England (reference number 12/EE/0038), and by the national and local research ethics committees in all participating non-UK countries.

For peer review only

References

1. Hearnshaw, S., et al., *Acute upper gastrointestinal bleeding in the UK: Patient characteristics, diagnoses and outcomes in the 2007 UK audit.* Gut, 2011;60(1327-35.
2. McPherson, S., et al., *Gastrointestinal Haemorrhage: Time to Get Control.* London, UK: National Confidential Enquiry into Patient Outcome and Death, 2015;
3. Crooks, C., T. Card, and J. West, *Reductions in 28-day mortality following hospital admission for upper gastrointestinal hemorrhage.* Gastroenterology, 2011;141(1):62-70.
4. Henderson, R. *Oesophageal Varices*. Gastroenterology 2016 [cited 2020 18 July]; Available from: https://patient.info/doctor/oesophageal-varices#.
5. Hwang, J.H., et al., *The role of endoscopy in the management of acute non-variceal upper GI bleeding.* Gastrointestinal Endoscopy, 2012;75(6):1132-1138.
6. Campbell, H.E., et al., *Costs and quality of life associated with acute upper gastrointestinal bleeding in the UK: cohort analysis of patients in a cluster randomised trial.* BMJ open, 2015;5(4):e007230.
7. Wallis, J.P., A.W. Wells, and C.E. Chapman, *Changing indications for red cell transfusion from 2000 to 2004 in the North of England.* Transfusion Medicine, 2006;16(6):411-417.
8. Villanueva, C., et al., *Transfusion Strategies for Acute Upper Gastrointestinal Bleeding.* New England Journal of Medicine, 2013;368(1):11-21.
9. Dunn, C.J. and K.L. Goa, *Tranexamic acid.* Drugs, 1999;57(6):1005-1032.
10. Vermylen, J., et al., *A double blind study of the effect of tranexamic acid in essential menorrhagia.* Thrombosis and Haemostasis, 1968;20(02):583-587.
11. Srinil, S. and U. Jaisamrarn, *Treatment of idiopathic menorrhagia with tranexamic acid.* JOURNAL-MEDICAL ASSOCIATION OF THAILAND, 2005;88(S1.
12. Wardrop, D., et al., *Antifibrinolytics (lysine analogues) for the prevention of bleeding in patients with haematological disorders.* Cochrane Database of Systematic Reviews, 2013;7):
13. Watterson, C. and N. Beacher, *Preventing perioperative bleeding in patients with inherited bleeding disorders.* Evidence-Based Dentistry, 2017;18(1):28-29.
14. Nutbeam, T., *In adult patients presenting as emergencies with upper gastrointestinal bleeding, does tranexamic acid decrease mortality?* African Journal of Emergency Medicine, 2015;5(2):85-92.
15. The CRASH-3 Trial Collaborators, *Effects of tranexamic acid on death, disability, vascular occlusive events and other morbidities in patients with acute traumatic brain injury (CRASH-3): a randomised, placebo-controlled trial.* The Lancet, 2019;394(10210):1713-1723.
16. Shakur, H., et al., *Effect of early tranexamic acid administration on mortality, hysterectomy, and other morbidities in women with post-partum haemorrhage (WOMAN): an international, randomised, double-blind, placebo-controlled trial.* The Lancet, 2017;389(10084):2105-2116.
17. Olldashi, F., et al., *Effects of tranexamic acid on death, vascular occlusive events, and blood transfusion in trauma patients with significant haemorrhage (CRASH-2): a randomised, placebo-controlled trial.* The Lancet, 2010;376(9734):23-32.
18. Roberts, I., D. Prieto-Merino, and D. Manno, *Mechanism of action of tranexamic acid in bleeding trauma patients: an exploratory analysis of data from the CRASH-2 trial.* Critical Care, 2014;18(6):685.
19. Roberts, I., et al., *Effects of a high-dose 24-h infusion of tranexamic acid on death and thromboembolic events in patients with acute gastrointestinal bleeding (HALT-IT): an international randomised, double-blind, placebo-controlled trial.* The Lancet, 2020;395(10241):1927-1936.
20. Hawkey, G., et al., *Drug treatments in upper gastrointestinal bleeding: value of endoscopic findings as surrogate end points.* Gut, 2001;49(3):372-379.
21. Cormack, F., et al., *Tranexamic acid in upper gastrointestinal haemorrhage.* The Lancet, 1973;301(7814):1207-1208.
22. Bennett, C., et al., *Tranexamic acid for upper gastrointestinal bleeding.* Cochrane Database of Systematic Reviews, 2014;11):
23. Twum-Barimah, E., et al., *Systematic review with meta-analysis: the efficacy of tranexamic acid in upper gastrointestinal bleeding.* Alimentary Pharmacology & Therapeutics, 2020;
24. National Institute for Health and Care Excellence, *Acute upper gastrointestinal bleeding in over 16s: management [CG141].* 2012;
25. Oakland, K., et al., *Diagnosis and management of acute lower gastrointestinal bleeding: guidelines from the British Society of Gastroenterology.* Gut, 2019;68(5):776.
26. Roberts, I., et al., *A high-dose 24-hour tranexamic acid infusion for the treatment of significant gastrointestinal bleeding: HALT-IT RCT.* Health Technol Assess, 2021;25(58):1-86.

For peer review only - http://bmjopen.bmj.com/site/about/guidelines.xhtml

27. National Institute for Health and Care Excellence, *Guide to the methods of technology appraisal.* National Institute for Health and Clinical Excellence (NICE) London, UK, 2018;
28. Williams, J., et al., *Cost-effectiveness analysis of tranexamic acid for the treatment of traumatic brain injury, based on the results of the CRASH-3 randomised trial: a decision modelling approach.* BMJ global health, 2020;5(9):
29. Office for National Statistics. *National life tables, UK: 2016 to 2018*. Available from: https://www.ons.gov.uk/peoplepopulationandcommunity/birthsdeathsandmarriages/lifeexpectancies/bulletins/nationallifetablesunitedkingdom/2016to2018.
30. Crooks, C.J., T.R. Card, and J. West, *Excess long-term mortality following non-variceal upper gastrointestinal bleeding: a population-based cohort study.* PLoS medicine, 2013;10(4):e1001437.
31. Jairath, V., et al., *Restrictive versus liberal blood transfusion for acute upper gastrointestinal bleeding (TRIGGER): a pragmatic, open-label, cluster randomised feasibility trial.* Lancet, 2015;386(9989):137-44.
32. Katz, S., et al., *Studies of the illness in the aged, the index of ADL: A standarsized measure of biological and psychological functioning.* JAMA, 1963;185(914-9.
33. Kind, P., et al., *Variations in population health status: results from a United Kingdom national questionnaire survey.* BMJ, 1998;316(
34. Royal Pharmaceutical Society. *British National Formulary*. 2019 01/08/2016]; Available from: https://about.medicinescomplete.com/publication/british-national-formulary/.
35. Curtis, L. and A. Burns. *Unit costs of health and social care 2018*. 2018; Available from: https://www.pssru.ac.uk/project-pages/unit-costs/unit-costs-2018/.
36. Guerriero, C., et al., *Cost-Effectiveness Analysis of Administering Tranexamic Acid to Bleeding Trauma Patients Using Evidence from the CRASH-2 Trial.* PLoS One, 2011;6(5):e18987.
37. Li, B., et al., *Tranexamic acid for treatment of women with post-partum haemorrhage in Nigeria and Pakistan: a cost-effectiveness analysis of data from the WOMAN trial.* The Lancet. Global health, 2018;6(2):e222-e228.
38. NHS Improvement. *National schedule of reference costs 2017/18*. Available from: https://webarchive.nationalarchives.gov.uk/20200501111106/https://improvement.nhs.uk/resources/reference-costs/.
39. NHS Blood and Transport. *NHS Blood and Transport Price List 2018/2019*. Available from: https://nhsbtdbe.blob.core.windows.net/umbraco-assets-corp/14810/price_list_bc_nhs_2018-19.pdf.
40. Ramagopalan, S.V., et al., *Costs of gastrointestinal bleeding events in atrial fibrillation: a UK Clinical Practice Research Datalink study.* Future cardiology, 2019;15(5):367-375.
41. Gayet-Ageron, A., et al., *Effect of treatment delay on the effectiveness and safety of antifibrinolytics in acute severe haemorrhage: a meta-analysis of individual patient-level data from 40 138 bleeding patients.* The Lancet, 2018;391(10116):125-132.

For peer review only - http://bmjopen.bmj.com/site/about/guidelines.xhtml

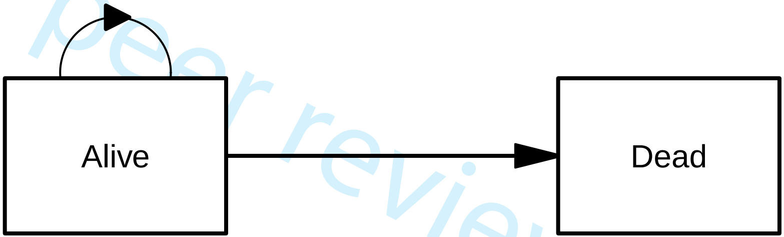

For peer review only - http://bmjopen.bmj.com/site/about/guidelines.xhtml

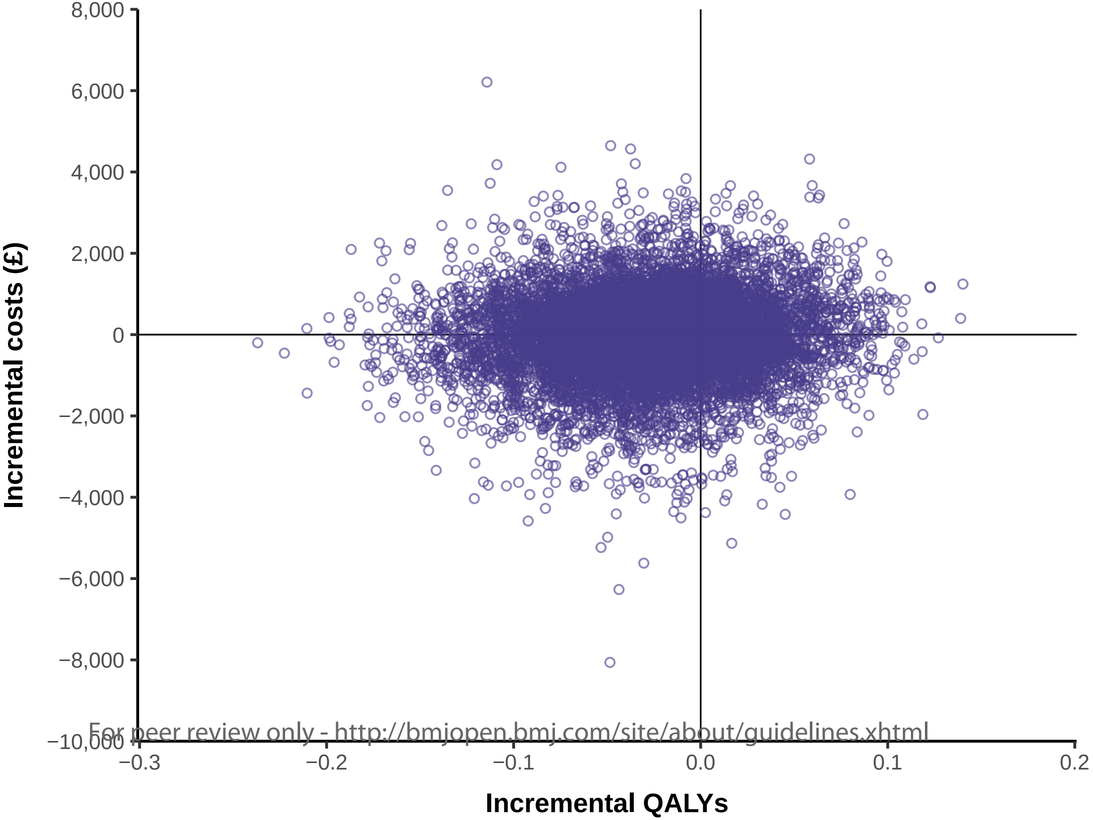

For peer review only - http://bmjopen.bmj.com/site/about/guidelines.xhtml

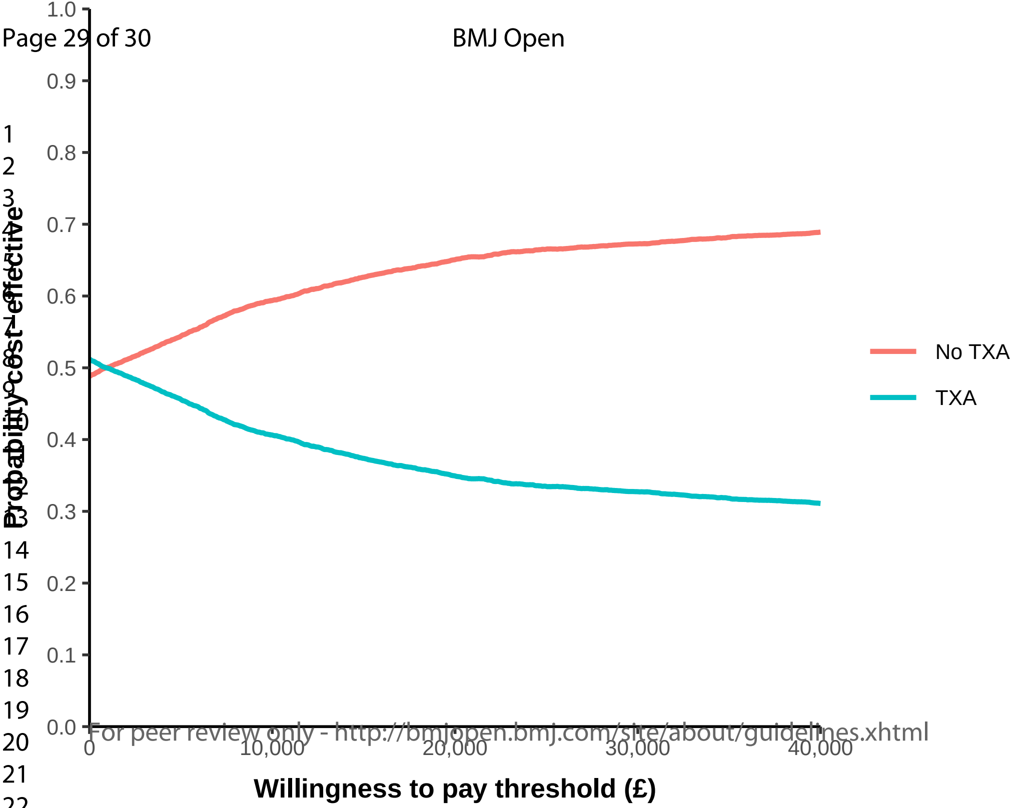

For peer review only - http://bmjopen.bmj.com/site/about/guidelines.xhtml

BMJ 2013;346:f1049 doi: 10.1136/bmj.f1049 (Published 25 March 2013)

Table

Table 1| CHEERS checklist—Items to include when reporting economic evaluations of health interventions

Section/item	Item No	Recommendation	Reported on page No/ line No
**Title and abstract**			
Title	1	Identify the study as an economic evaluation or use more specific terms such as "cost-effectiveness analysis", and describe the interventions compared.	P1 L1-3
Abstract	2	Provide a structured summary of objectives, perspective, setting, methods (including study design and inputs), results (including base case and uncertainty analyses), and conclusions.	P3
**Introduction**			
Background and objectives	3	Provide an explicit statement of the broader context for the study.	P6 L13-17
		Present the study question and its relevance for health policy or practice decisions.	P6 L19-27
**Methods**			
Target population and subgroups	4	Describe characteristics of the base case population and subgroups analysed, including why they were chosen.	P7 L3-4, L12-14, P7 L26-27
Setting and location	5	State relevant aspects of the system(s) in which the decision(s) need(s) to be made.	P7 L6
Study perspective	6	Describe the perspective of the study and relate this to the costs being evaluated.	P7 L6
Comparators	7	Describe the interventions or strategies being compared and state why they were chosen.	P7 L3-5
Time horizon	8	State the time horizon(s) over which costs and consequences are being evaluated and say why appropriate.	P7 L5-6
Discount rate	9	Report the choice of discount rate(s) used for costs and outcomes and say why appropriate.	P7 L8-9
Choice of health outcomes	10	Describe what outcomes were used as the measure(s) of benefit in the evaluation and their relevance for the type of analysis performed.	P7 L7-8
Measurement of effectiveness	11a	*Single study-based estimates:* Describe fully the design features of the single effectiveness study and why the single study was a sufficient source of clinical effectiveness data.	P8 L12-18
	11b	*Synthesis-based estimates:* Describe fully the methods used for identification of included studies and synthesis of clinical effectiveness data.	
Measurement and valuation of preference based outcomes	12	If applicable, describe the population and methods used to elicit preferences for outcomes.	P8 L22-25
Estimating resources and costs	13a	*Single study-based economic evaluation:* Describe approaches used to estimate resource use associated with the alternative interventions. Describe primary or secondary research methods for valuing each resource item in terms of its unit cost. Describe any adjustments made to approximate to opportunity costs.	
	13b	*Model-based economic evaluation:* Describe approaches and data sources used to estimate resource use associated with model health states. Describe primary or secondary research methods for valuing each resource item in terms of its unit cost. Describe any adjustments made to approximate to opportunity costs.	P9 L6 - P10 L23
Currency, price date, and conversion	14	Report the dates of the estimated resource quantities and unit costs. Describe methods for adjusting estimated unit costs to the year of reported costs if necessary. Describe methods for converting costs into a common currency base and the exchange rate.	L7 L6-7
Choice of model	15	Describe and give reasons for the specific type of decision-analytical model used. Providing a figure to show model structure is strongly recommended.	P7 L17-19 Figure 1
Assumptions	16	Describe all structural or other assumptions underpinning the decision-analytical model.	P7 L26-27, P9 L10-15, P10 L18-23 (in methods)
Analytical methods	17	Describe all analytical methods supporting the evaluation. This could include methods for dealing with skewed, missing, or censored data; extrapolation methods; methods for pooling data; approaches to validate or make adjustments (such as half cycle corrections) to a model; and methods for handling population heterogeneity and uncertainty.	P8 L12-19 P8 L26-31 P10 L26 - P11 L11
**Results**			
Study parameters	18	Report the values, ranges, references, and, if used, probability distributions for all parameters. Report reasons or sources for distributions used to represent uncertainty where appropriate. Providing a table to show the input values is strongly recommended.	Table 1, Table 2 P10 L26 - P11 L11
Incremental costs and outcomes	19	For each intervention, report mean values for the main categories of estimated costs and outcomes of interest, as well as mean differences between the comparator groups. If applicable, report incremental cost-effectiveness ratios.	P12 L3-8, Table 3
Characterising uncertainty	20a	*Single study-based economic evaluation:* Describe the effects of sampling uncertainty for the estimated incremental cost and incremental effectiveness parameters, together with the impact of methodological assumptions (such as discount rate, study perspective).	

BMJ: first published as 10.1136/bmj.f1049 on 25 March 2013. Downloaded from http://www.bmj.com/ on 21 December 2021 by guest. Protected by copyright.

No commercial reuse: See rights and reprints http://www.bmj.com/permissions Subscribe: http://www.bmj.com/subscribe

(continued)

Section/item	Item No	Recommendation	Reported on page No/ line No
	20b	*Model-based economic evaluation:* Describe the effects on the results of uncertainty for all input parameters, and uncertainty related to the structure of the model and assumptions.	Figure 2, Figure 3, Table 4
Characterising heterogeneity	21	If applicable, report differences in costs, outcomes, or cost-effectiveness that can be explained by variations between subgroups of patients with different baseline characteristics or other observed variability in effects that are not reducible by more information.	N/A
**Discussion**			
Study findings, limitations, generalisability, and current knowledge	22	Summarise key study findings and describe how they support the conclusions reached. Discuss limitations and the generalisability of the findings and how the findings fit with current knowledge.	P14-15
**Other**			
Source of funding	23	Describe how the study was funded and the role of the funder in the identification, design, conduct, and reporting of the analysis. Describe other non-monetary sources of support.	P23 L10-11
Conflicts of interest	24	Describe any potential for conflict of interest of study contributors in accordance with journal policy. In the absence of a journal policy, we recommend authors comply with International Committee of Medical Journal Editors recommendations.	P23 L7-8

For consistency, the CHEERS statement checklist format is based on the format of the CONSORT statement checklist

BMJ: first published as 10.1136/bmj.f1049 on 25 March 2013. Downloaded from http://www.bmj.com/ on 21 December 2021 by guest. Protected by copyright.

No commercial reuse: See rights and reprints http://www.bmj.com/permissions Subscribe: http://www.bmj.com/subscribe

BMJ Open

An economic evaluation of tranexamic acid for the treatment of acute gastrointestinal bleeding: A cost-effectiveness analysis using data from the HALT-IT randomised controlled trial

Journal:	*BMJ Open*
Manuscript ID	bmjopen-2021-060505.R1
Article Type:	Original research
Date Submitted by the Author:	16-Mar-2022
Complete List of Authors:	Bazeer, Nuha; London School of Hygiene & Tropical Medicine, London, UK and London School of Economics and Political Science, London, UK<br>Miners, Alec; London School of Hygiene & Tropical Medicine, Department of Health Services Research and Policy<br>Roberts, Ian; London School of Hygiene & Tropical Medicine, Clinical Trials Unit<br>Shakur-Still, Haleema; London School of Hygiene & Tropical Medicine, Clinical Trials Unit<br>Jairath, Vipul; Western University, Department of Medicine, Division of Gastroenterology<br>Williams, Jack; London School of Hygiene & Tropical Medicine, Department of Health Services Research and Policy
**Primary Subject Heading**:	Health economics
Secondary Subject Heading:	Gastroenterology and hepatology, Haematology (incl blood transfusion)
Keywords:	GASTROENTEROLOGY, Bleeding disorders & coagulopathies < HAEMATOLOGY, HEALTH ECONOMICS

SCHOLARONE™
Manuscripts

BMJ

I, the Submitting Author has the right to grant and does grant on behalf of all authors of the Work (as defined in the below author licence), an exclusive licence and/or a non-exclusive licence for contributions from authors who are: i) UK Crown employees; ii) where BMJ has agreed a CC-BY licence shall apply, and/or iii) in accordance with the terms applicable for US Federal Government officers or employees acting as part of their official duties; on a worldwide, perpetual, irrevocable, royalty-free basis to BMJ Publishing Group Ltd ("BMJ") its licensees and where the relevant Journal is co-owned by BMJ to the co-owners of the Journal, to publish the Work in this journal and any other BMJ products and to exploit all rights, as set out in our licence.

The Submitting Author accepts and understands that any supply made under these terms is made by BMJ to the Submitting Author unless you are acting as an employee on behalf of your employer or a postgraduate student of an affiliated institution which is paying any applicable article publishing charge ("APC") for Open Access articles. Where the Submitting Author wishes to make the Work available on an Open Access basis (and intends to pay the relevant APC), the terms of reuse of such Open Access shall be governed by a Creative Commons licence – details of these licences and which Creative Commons licence will apply to this Work are set out in our licence referred to above.

Other than as permitted in any relevant BMJ Author's Self Archiving Policies, I confirm this Work has not been accepted for publication elsewhere, is not being considered for publication elsewhere and does not duplicate material already published. I confirm all authors consent to publication of this Work and authorise the granting of this licence.

For peer review only

An economic evaluation of tranexamic acid for the treatment of acute gastrointestinal bleeding: A cost-effectiveness analysis using data from the HALT-IT randomised controlled trial

Authors:

Nuha Bazeer[1] (0000-0002-4851-8402)

Alec Miners[2] (0000-0003-1850-1463)

Ian Roberts[3] (0000-0003-1596-6054)

Haleema Shakur-Still[3] (0000-0002-6511-109X)

Vipul Jairath[4] (0000-0002-1092-0033)

Jack Williams[2] (0000-0002-1331-387X)

1 London School of Hygiene & Tropical Medicine, London, UK and London School of Economics and Political Science, London, UK

2 Department of Health Services Research and Policy, London School of Hygiene & Tropical Medicine, London, UK

3 Clinical Trials Unit, London School of Hygiene & Tropical Medicine, London, UK

4 Department of Medicine, Division of Gastroenterology, Western University, London, Canada

For peer review only

Corresponding author:
Jack Williams
London School of Hygiene & Tropical Medicine
Room 142, 15-17 Tavistock Place, London, WC1H 9SH
Jack.Williams@lshtm.ac.uk

For peer review only

For peer review only - http://bmjopen.bmj.com/site/about/guidelines.xhtml

Abstract

Objective: To perform an economic evaluation of tranexamic acid (TXA) versus no TXA, in additional to current clinical practice, for acute gastrointestinal bleeding, using the results of the HALT-IT trial, a large randomised controlled trial which included 11,937 patients.

Design: A cost-effectiveness modelling analysis, performed over a lifetime time horizon.

Setting: The analysis was performed from a UK health service perspective

Participants: The model includes adults with acute gastrointestinal bleeding.

Outcomes measures: The model reports costs in Great British Pounds (GBP) in 2021 and outcomes as life years (LYs) and quality-adjusted life years (QALYs). Cost-effectiveness was evaluated using incremental cost-effectiveness ratios (ICERs), reported as the cost per QALY gained.

Methods: A Markov model was developed to calculate the overall costs and health outcomes of TXA administration versus no TXA. The model used data of the treatment effectiveness from the HALT-IT trial, which showed that TXA administration for acute gastrointestinal bleeding did not reduce all-cause mortality (RR 1.03, 95% CI 0.92; 1.16) compared to no TXA. Data on health-related quality of life, costs, and long-term mortality risks were derived from the literature. Costs and effects are discounted at 3.5% per annum.

Results: TXA was associated with marginally fewer LYs and QALYs, and lower costs, than treatment without TXA. The ICER associated with no TXA was £1,576 per LY gained and £2,209 per QALY gained. No TXA was 64% likely to be cost-effective at a £20,000 willingness to pay threshold, whilst TXA was 36% likely to be cost-effective.

Conclusion: Though inexpensive, TXA administration for patients with acute gastrointestinal bleeding is unlikely to be cost-effective.

For peer review only - http://bmjopen.bmj.com/site/about/guidelines.xhtml

Strengths and limitations of this study

- This is the first economic evaluation of tranexamic acid for the treatment of acute gastrointestinal bleeding
- Data for the treatment effect comes from the HALT-IT trial, which included 11,937 patients receiving either tranexamic acid or placebo.
- The cost-effectiveness model extrapolates the impact of tranexamic acid treatment over a lifetime time horizon, as recommended by the National Institute for Health and Care Excellence, for UK based economic evaluations
- The HALT-IT trial data only reports outcomes up to 28 days following the onset of acute gastrointestinal bleeding, and therefore the model only accounts for treatment effects observed within this 28 day trial period.

For peer review only

For peer review only - http://bmjopen.bmj.com/site/about/guidelines.xhtml

Introduction

Acute gastrointestinal bleeding (AGIB) refers to bleeding arising from the upper or lower gastrointestinal tract. Acute upper gastrointestinal bleeding (AUGIB) occurs more commonly than acute lower gastrointestinal bleeding (ALGIB) and has a higher case fatality rate of up to 10% [1]. AUGIB is a common medical emergency and results in approximately 85,000 cases each year within the United Kingdom, with around 4,000 AUGIB related deaths annually [2]. Thus, AUGIB presents a significant health concern with an estimated incidence of 133 cases per 100,000 population, accounting for 8% of all acute hospital admissions [2]. The leading causes of AUGIB have been recorded as peptic ulcers and esophagogastric varices [1, 3]. While peptic ulcers are often associated with long-term use of non-steroid anti-inflammatory drugs (NSAIDs), esophagogastric varices are typically complications of liver cirrhosis [4]. Comparisons of national AUGIB audits in the United Kingdom carried out in 1994 and 2007 have shown improved outcomes amongst patients, likely through advancements in endoscopic and some pharmacological therapies [1]. However, over the past two decades, there have been no significant changes to mortality associated with AUGIB, and mortality amongst older patients with other comorbidities remains high at 10% [2, 5]. Within the UK, annual in-hospital costs for all AUGIB cases have been estimated as £155.5 million, of which £12.6 million accounts for blood product transfusions (BPT) alone [6]. As the second most common medical reason for transfusion, GI bleeding accounts for 14% of all BPT within the UK, with inappropriate use recorded in 20% of cases [7, 8].

Tranexamic acid (TXA) is an anti-fibrinolytic agent which reduces fibrinolysis and inhibits the breakdown of blood clots [9]. It's used to treat women with heavy menstrual bleeding (menorrhagia) and people with haemophilia during dental extractions [10-13]. The efficacy of TXA has also been demonstrated in reducing blood loss during surgical operation trials [9]. It is widely available, relatively inexpensive, and can be administered through oral or intravenous formulations, without specialist monitoring [14]. Large, multi-centre, randomised controlled trials (RCTs) such as the WOMAN and CRASH-2 and -3 trials have shown TXA to significantly reduce bleeding mortality amongst patients at risk of postpartum haemorrhage and bleeding trauma patients respectively [15-17]. However, the timing of TXA administration is of particular importance, with no evidence of a benefit upon mortality when administered more than three hours after injury in both WOMAN and CRASH-2 and -3 trials. [16, 18]. Unlike postpartum haemorrhage or brain injury however, the onset of a gastrointestinal bleed may be less discernible, with presentation often delayed due to symptoms only becoming

For peer review only - http://bmjopen.bmj.com/site/about/guidelines.xhtml

visible when most severe. This was observed within the HALT-IT trial, with over 80% of patients presenting more than three hours after the onset of bleeding [19].

Previously conducted smaller RCTs in the UK have found no evidence of a difference in mortality, rebleeding, and the requirement of surgery [20, 21]. However, a 2014 Cochrane systematic review and meta-analysis of RCTs comparing TXA with placebo for AUGIB estimated large reductions in all-cause mortality, with a pooled risk ratio (RR) of 0.6 (95% CI, 0.42–0.87) across 8 studies with 1701 participants [22]. However, the mortality benefit of TXA was uncertain when excluding trials with a high risk of bias. For this reason, the authors concluded that tranexamic acid cannot be recommended for routine clinical practice. An updated systematic review and meta-analysis found similar findings, with a risk ratio of 0.59 (95% CI 0.43-0.82), and also concluded that high-quality RCTs were needed [23].

TXA is not currently recommended within UK guidelines for AUGIB or ALGIB [24]. National Institute for Health and Care Excellence (NICE) guidelines for AUGIB refer to limited evidence of an effect of TXA on mortality, but do not include recommendations for TXA in the absence of UK marketing authorisation for this indication. Similarly, guidelines from the British Society of Gastroenterology state that the mortality benefit of TXA is uncertain when excluding trials with a low risk of bias, suggesting recommendations are made following the HALT-IT trial [25].

The HALT-IT trial was a large multinational RCT that compared TXA versus placebo, in addition to current practice, in 11,937 people with AGIB [19]. The trial did not find evidence that TXA significantly reduces any of the key outcome measures, such as death due to bleeding within 5 days of randomisation (RR 0.99; 95% CI 0.82; 1.18) or all-cause mortality (RR 1.03 95% CI 0.92; 1.16). This means by default, even if relatively inexpensive, TXA is unlikely to represent value for money in this indication. However, there is still a need to perform an economic evaluation based on the HALT-IT results, in order to establish the level of uncertainty about this conclusion and to assess the extent to which only including the results from the systematic review would have impacted the cost-effectiveness estimates. The full results of the HALT-IT trial, including the economic results, are available in a separate National Institute of Health Research report [26].

For peer review only - http://bmjopen.bmj.com/site/about/guidelines.xhtml

For peer review only

Methods

Analysis

The base case economic analysis assessed the cost-effectiveness of treating patients identified as sustaining an AGIB with TXA versus no TXA, in addition to current practice, using estimates of treatment effect from the HALT-IT trial. In line with NICE guidelines for health technology appraisals, A cost-effectiveness model was developed in Microsoft Excel and was analysed over a lifetime time horizon, from a UK NHS health service perspective [27]. All costs are reported in 2021 Great British Pounds, with costs inflated using an NHS cost inflation index [28]. Health outcomes were assessed as life years (LYs), and quality-adjusted life years (QALYs). All future costs and health outcomes were discounted at 3.5% per annum, as per NICE guidance [27]. We report the results as incremental cost-effectiveness ratios (ICERs) by diving the incremental costs by the incremental outcomes. We also report results using incremental net monetary benefits (INMBs), by converting incremental outcomes in monetary values based on the willingness to pay threshold, and then subtracting the incremental costs. This means that positive INMB represents a cost-effective scenario, whilst a negative INMB does not. A willingness to pay threshold of £20,000 per QALY has been used to determine cost-effectiveness, in line with NICEs Technology Appraisals recommendations [27].

The HALT-IT trial included a number of sub-group analyses, but as no treatment effects were observed amongst subgroups, they were not considered in the model. Whilst the trial consisted mostly of patients with AUGIB (89%), the model population includes both AUGIB and ALGIB, as per the overall trial population.

Model Structure

An existing Markov model of TXA following traumatic brain injury was adapted to estimate the cost-effectiveness of TXA for patients with AGIB [29]. The model consists of two health states *alive* and *dead*; as shown in Figure 1. People with AGIB enter the model in the *alive* health state. They progress to the *dead* health state over the initial 28-day period (the HALT-IT trial period) using daily data taken directly from the trial on all-cause deaths from the placebo arm of HALT-IT; this includes data for all UK and non-UK participants. For the remaining 337 days of the first year, the daily probability of death in the no-TXA arm was estimated using UK general population age-related mortality statistics [30], adjusted using a standardised mortality ratio (SMR) to reflect the long-term risk of excess death associated with surviving an AGIB [31]. In the base case, this SMR is

For peer review only - http://bmjopen.bmj.com/site/about/guidelines.xhtml

applied for the entire time horizon. After this initial year period, the model uses an annual cycle length, and applies a probability of death based on UK all-cause mortality data, combined with the SMR. In line with the HALT-IT trial, the mean age of people entering the model was assumed to be 58.1 years (Table 1).

The SMR associated with the risk of death following an AGIB compared to that of the general population was derived from a study by Crooks et al (2013) [31]. It linked longitudinal data from the UKs Hospital Episodes Statistics dataset, General Practice Research Database and Office of National Statistics death register for people with non-variceal bleeds between 1997 and 2010. Controls were matched by age, sex, practice, and year. The study reported the risk of death was highest in the first year following an AGIB (SMR 5.21 95% CI 4.25; 6.38), and remained elevated from year 2 onwards (SMR 1.74 95% CI 1.42; 2.13).

Treatment Effect

The HALT-IT trial results for the primary outcome measure, death due do bleeding within 5 days from randomisation, was similar in both treatment arms (RR 0.99; 95% CI 0.82; 1.18), although the mean estimate slightly favoured TXA.[19] However, as the risks of venous thromboembolic events (RR 1.85; 95% CI 1.15; 2.98), death from malignancy (RR 1.63; 95% CI 1.10; 2.42), and seizure (RR 1.73; 95% CI 1.03; 2.93) were higher in the TXA- than placebo-group, the base case RR of all-cause mortality was used to estimate the overall effect of TXA treatment on survival (RR 1.03 95% CI 0.92; 1.16), and is shown in Table 1. This RR is based on all trial participants and is not UK specific since the subgroup analysis did not reveal any differences in treatment effect by country income level.

Utilities

Utility data were not collected as part of HALT-IT, therefore values were sourced from the existing literature. Only one directly relevant study could be identified – the relatively recent TRIGGER RCT [32]. The EQ-5D-3L questionnaire was administered at discharge or day-28, with an estimated utility of 0.735 (95% CI 0.70; 0.77) for those surviving an AGIB (Table 1) [6]. In line with previous economic evaluations of TXA, this single utility value of 0.735 was applied to all people who were alive at day-28, both during the initial 28-day period and at all times thereafter until death, in both treatment arms [29]. Note that functional status was measured in HALT-IT using the Katz Index of Independence in Activities of Daily Living [33], with the scores similar in both arms (score of 5.5 for both).

For peer review only - http://bmjopen.bmj.com/site/about/guidelines.xhtml

Since the TRIGGER RCT population has a slightly lower level of co-morbidities and complications amongst participants compared to the HALT-IT population, we considered the impact of a much lower utility value (0.5) for all surviving patients following AGIB.

An age adjustment was also applied to the utility value each cycle using the decrements reported by Kind et al., so that overall values declined with age [34].

Costs

Treatment costs

The resource use associated with providing TXA treatment were derived from the HALT-IT trial and by making a number of assumptions. The total TXA dose (4g) included a loading- and maintenance-dose, for which the total cost was derived from the British National Formulary (£12 per patient) [35]. The cost of equipment for treatment was assumed to include a needle (£0.05), syringe (£0.07), sodium chloride infusion bag (£0.59) and isotonic IV solution (£2.96) [35]. To account for the administration time of TXA, hourly staff costs were taken from the Personal Social Services Research Unit for 2021 [28]. The model assumed that a Band 5 nurse would take 21 minutes to administer TXA (£14.35), following the treatment administration and expert guidance used within the economic evaluations of the CRASH [29, 36] and WOMAN trials [37]. This led to a total TXA treatment and administration cost of £30.01 per person (Table 2).

Inpatient stay costs

Information from HALT-IT was used to derive the costs of inpatient stays during the initial 28 day trial period. The total mean lengths of stay recorded by people receiving TXA and placebo were 5.83 and 5.80 days respectively (Table 2). In both treatment arms, 0.4 of these days were spent in intensive care units (ICU). These lengths of stay were multiplied by NHS Reference Costs for non-elective stays to derive mean hospital costs for TXA and no TXA of £2,471 and £2,462 per person respectively [38].

Procedures and Transfusions

While in hospital during the 28-day trial period, participants underwent a number of procedures, including endoscopy, surgery and radiological interventions (Table 2). The cost of these procedures were calculated for each trial arm using the probability of occurrence from the HALT-IT trial multiplied by the NHS

For peer review only - http://bmjopen.bmj.com/site/about/guidelines.xhtml

Reference Costs [38]. The mean per person procedural costs for the TXA and no TXA treatment options were £2,389 and £2,437 respectively.

People also received transfusions of blood / red blood cells, fresh frozen plasma and platelets whilst in hospital (Table 2). Information from HALT-IT on the frequency and the mean number of units for each transfusion type were combined with unit costs from the NHS Blood and Transfusions price list to generate overall costs [39]. The mean per person transfusion costs for the TXA and no TXA treatment options were £253 and £264 respectively.

Whilst the model did not explicitly account for adverse events during the trial period, the costs of these adverse events are included in the hospital length of stay, procedures and transfusions that occur during the hospital stay.

Post discharge costs

The HALT-IT and TRIGGER trials observed participants for a maximum of 28-days, therefore longer-term care costs were sourced from the literature. A specific study related to AGIB could not be identified. However, a recent UK study by Ramagopalan et al. quantified the costs of gastrointestinal bleeding among people with nonvalvular atrial fibrillation (NVAF), who were and were not experiencing a bleed, and used a difference in difference approach to calculate the costs attributable to a gastrointestinal bleed (cases, n=7,753 and controls, n=7,753) [40].

The study used NHS reference costs and calculated the annual costs of caring for people with a bleed to be £4,350, £1,980 and £1,938 in the first second- and third-years post-bleed (Table 2). In the absence of other information, the reported year three costs were inputted into the model for years four onwards, although they were assumed to be one third the original amount. The standard errors that the costing study reports could not be easily incorporated into our analysis (the statistical approach used allowed costs to be negative), therefore they were assumed to be 50% of the mean value in all instances, which is thought to reflect a relatively large degree of uncertainty around the mean estimates. The importance of these assumptions was assessed in the sensitivity analysis.

Sensitivity Analysis

Various one-way deterministic sensitivity analyses were undertaken to assess the robustness of the results to alternative assumptions. In line with NICE guidelines, one-way DSA was conducted on the discount rate for

For peer review only - http://bmjopen.bmj.com/site/about/guidelines.xhtml

both costs and health outcomes, using values of 0% and 6%. It was also conducted on a range of scenarios considering the extrapolation of the monitoring costs, health-related quality of life for AGIB survivors, and TXA treatment effect.

To simultaneously investigate the effect of parameter uncertainty, probabilistic sensitivity analysis (PSA) was also undertaken using 10,000 Monte Carlo simulations; the parameter distributions are specified in Table 1 and Table 2. The probabilistic results are presented on a cost-effectiveness plane, and as a cost-effectiveness acceptability curve (CEAC). Parameters that were excluded from the PSA are not assigned a distribution type in the tables.

A scenario analysis was performed to consider the economic results that would have been generated prior to the availability of the results of TXA from the HALT-IT trial. This analysis was performed primarily to demonstrate the differences in decision making when using low or moderate quality evidence to populate the economic model, particularly from trials with a high risk of bias. In this analysis the 28-day mortality risk was 0.084 (71/850) in the control group, whilst the TXA all-cause mortality RR was 0.60 (95% CI: 0.42-0.87), as per the Cochrane analysis [22].

Patient and Public Involvement

Patient and public input was used to inform the HALT-IT trial procedures, including study information sheets and consent forms.[26] Patients stated that the research should account for quality of life issues in additional to the treatment efficacy and safety, which this economic analysis does. There was no patient or public involvement specifically in the design of the economic model.

For peer review only - http://bmjopen.bmj.com/site/about/guidelines.xhtml

Results

Base-Case Analysis

The base case results are shown in Table 3. In both the deterministic and probabilistic analyses TXA was associated with marginally fewer LYs and QALYs, and marginally lower costs, than treatment without TXA. The deterministic QALYs and costs associated with TXA and no-TXA were 8.58 and 8.61, and £18,155 and £18,220 respectively. This meant that providing TXA was £64.30 less costly, and resulted in 0.041 fewer QALYs, per person treated.

Since TXA was less costly and less effective in the base case analysis compared to placebo, the ICERs can be reported as £1,576 saved for each LY lost, and £2,209 saved for each QALY lost. Since we value a QALY at £20,000 using the NICE willingness to pay threshold, this does not represent a cost-effective option, because the value of the health outcomes foregone are higher than the cost savings which occur. Alternatively the results can be expressed as no-TXA versus TXA, with no-TXA costing £1,576 per LY gained and £2,209 per QALY gained, meaning no-TXA is preferred at the NICE willingness to pay threshold. Either way, providing TXA is not cost-effective. The deterministic and mean probabilistic results are very similar.

The lower mean health outcomes for TXA are driven by the fact the mean risk ratio for all-cause mortality (i.e. the relative treatment effect) slightly favours the no-TXA treatment arm (RR 1.03). The higher incremental costs associated with no-TXA administration are largely driven by the fact people who did not receive TXA live for marginally longer than those who received TXA, thus they incur slightly higher post discharge costs, and marginally higher costs for procedures and transfusions, exceeding the additional low cost of TXA administration.

Probabilistic Sensitivity Analysis

The cost-effectiveness plane shows that the PSA simulations are clustered around the origin and distributed across all four cost-effectiveness quadrants (Figure 2). This is because the point estimate for the risk ratio of all-cause mortality is only slightly above one, and is included within its confidence interval (RR 1.03 95% CI 0.92; 1.16). Therefore, there is little to choose between the two treatment options in terms of outcomes. The same is also true of the relatively low cost of TXA administration (£30.01 per person), meaning that the majority of the costs incurred are the hospital costs (hospital stay, procedures and transfusions) and post-discharge costs.

For peer review only - http://bmjopen.bmj.com/site/about/guidelines.xhtml

The CEAC (Figure 3) shows that at any willingness to pay for an additional QALY above £2,000, no-TXA is likely to be the more cost-effective option. At the NICE specified willingness to pay threshold levels of £20,000 and £30,000 per additional QALY, TXA has a 36% and 33% probability of being the most cost-effective option, respectively. Note that the base case CEAC does not indicate that there is a 0% probability TXA is the most cost-effective treatment option, irrespective of the willingness to pay value. TXA treatment produced more QALYs at lower cost (i.e. it was the 'dominant' treatment option) in 15% of simulations. Rerunning the PSA assuming the post discharge costs had a standard error of 20%, instead of 50%, of their mean value, had a negligible impact on the probability of TXA being cost-effective.

Deterministic Sensitivity Analysis

Various deterministic one-way sensitivity analyses are shown Table 4. In all but one instance the results show that TXA is not cost-effective, and the ICER was very robust to alternative model parameter and structural assumptions. The exception is when the risk ratio of all-cause mortality is reduced to 0.92 (the lower bound of the 95% CI). In this instance, TXA produces marginally better health outcomes at slightly higher costs than no-TXA, producing an ICER of £1,227 per QALY gained if TXA is used instead of no TXA. Note that even when the cost of TXA and its administration was set to £0 instead of the base case value of £30.01, it is not cost-effective.

Meta-analysis scenario analysis

When parameterising the model with the mortality risk and all-cause mortality treatment effect reported from a meta-analysis containing multiple studies at risk of bias, prior to the availability of the HALT-IT trial, TXA becomes highly cost-effective, with an ICER of £1,040 per LY gained and £1,459 per QALY gained. In the probabilistic analysis, TXA was 99.5% likely to be cost-effective at the £20,000 per QALY threshold.

For peer review only

For peer review only - http://bmjopen.bmj.com/site/about/guidelines.xhtml

Discussion

The results from the base case analysis suggest that the costs and outcomes of treating people with AGIB with and without TXA, as enrolled and treated in the HALT-IT trial, are very similar. Despite being a relatively inexpensive intervention, TXA is unlikely to be a cost-effective use of NHS resources [19], and no-TXA is likely to be the most cost-effective option. These results are in line with our prior expectations given the additional, albeit modest, cost of providing TXA, which did not reduce mortality. However, the base case probabilistic analysis showed that there is a 36% chance TXA is the most cost-effective option at £20,000 per QALY thresholds, since there was uncertainty around the all-cause mortality treatment effect, with the risk ratio confidence interval ranging from 0.92 to 1.16.

As with all decision models, our analysis includes a number of parameter and structural assumptions. Whilst the analysis calculated the hospital costs associated with the hospital stay and procedures performed, the model did not explicitly include the long-term impacts of adverse events. Instead, the data used to extrapolate the costs and effects following the 28-day trial period were assumed to include AGIB patients with similar co-morbidities and complications following their GI bleed.

However, extensive sensitivity analysis showed that the parameter values for the costs and utilities following AGIB were generally unimportant in terms of changing the conclusion that TXA is unlikely to be cost-effective in this indication. The only sensitivity analysis in which TXA was cost-effective was when it was assumed to reduce mortality. Given that TXA resulted in marginally lower costs than no TXA (due to marginally lower hospital costs during the trial period), it would be cost-effective if it resulted in any all-cause mortality benefit. Given this, if future research identified a modest treatment effect for a subgroup of AGIB patients, then TXA would likely be cost-effective in this group.

To the best of our knowledge, no other cost-effectiveness analyses of TXA for people with AGIB have been reported. However, economic evaluations based on the series of CRASH RCTs and WOMAN RCT have concluded TXA is highly cost-effective following trauma injury and post-partum haemorrhage [15, 16, 29, 37]. The dose of TXA used in the HALT-IT trial is higher than that used in the previous studies, but this increased cost is negligible. The reason for these differences in the cost-effectiveness results is driven by the mortality benefit associated with TXA in other indications, which was not observed the HALT-IT trial population, or in any sub-group analyses. Of considerable importance and highlighted within the WOMAN and CRASH-3 studies was the time to treatment, with earlier treatment (within 3 hours of onset of bleeding) associated with improved

For peer review only - http://bmjopen.bmj.com/site/about/guidelines.xhtml

outcomes [41]. In AGIB patients, it is often difficult to identify the onset of bleeding, and presentation is often delayed. In the HALT-IT trial, 80% of participants received treatment more than 3 hours after the suspected onset of bleeding. Furthermore, patients with AGIB are older, with more comorbidities [19].

One of the main strengths of this analysis is that it is the first economic evaluation of TXA for people with AGIB based on the results of a large RCT. The results from HALT-IT are in contrast to previous meta-analyses of small trials, in which TXA was estimated to result in a large reduction in all-cause mortality in people with AUGIB, when including trials at risk of bias [22, 23]. A scenario analysis using these meta-analysis results found that, had an economic evaluation using the results of this meta-analysis been performed prior to the HALT-IT trial, the results would have suggested TXA was almost certainly cost-effective, with little uncertainty, and indicated little value in performing additional research. With hindsight, we believe the authors of these analyses were correct to conclude that their findings were insufficient to recommend the routine use of TXA in clinical practice, since this would have potentially led to poorer outcomes at a health systems level and potentially impacted on HALT-IT recruitment. For future health economic analyses, we suggest that researchers should be cautious in their use of meta-analyses that include trials considered susceptible to error or bias, which could subsequently result in misleading policy recommendations.

In carrying out an economic evaluation with robust sensitivity analysis, our findings emphasise the importance of large RCTs designed to ascertain the effectiveness of an intervention, and the importance of an economic analysis using high quality RCT data, to assess the cost-effectiveness of low-cost interventions.

Conclusion

Building on the evidence provided from the largest RCT in patients with AGIB, the results of this analysis have shown TXA administration for patients with AGIB is, on balance, unlikely to be cost-effective at UK WTP thresholds, and should not be recommended based on the current economic evidence. However, there remains uncertainty around this conclusion, with TXA being the most cost-effective treatment option in approximately one-third of simulations. This is because TXA would be cost-effective if it resulted in any mortality benefit, and the confidence interval for the all-cause mortality treatment effect observed in the HALT-IT trial (0.92-1.16), does not rule out such a finding. These results also highlight that if a modest treatment effect for a subset of patients can be identified in the future, then TXA has considerable scope to be cost-effective for this group.

For peer review only - http://bmjopen.bmj.com/site/about/guidelines.xhtml

Tables

Table 1: Base case model input parameters

Parameter	Value	95% CIs	Distribution	Source
Age at model start	*58.1*	-	-	HALT-IT
SMR year 1	*5.21*	4.25; 6.38	Log normal	Crooks et al [31]
SMR year 2	*1.74*	1.42; 2.13	Log normal	Crooks et al [31]
RR of all-cause mortality	*1.03*	0.92; 1.16	Log normal	HALT-IT
28-day probability of death†	*0.092*	0.084; 0.099	Beta	HALT-IT
Utility AGIB survivor	*0.735*	0.70; 0.77	Beta	Campbell et al [6]
Utility decrements by age (years)				Kind et al [34]
55-64	*0*	-	-	
65-74	*0.02*	-	-	
≥75	*0.07*	-	-	
Utility UK general population	*By age*	-	-	Kind et al [34]
All-cause mortality	*By age*	-	-	ONS [30]
Discount rate costs	*3.5%*	-	-	NICE [27]
Discount rate QALYs	*3.5%*	-	-	NICE **[27]**

†The 28-day risk of death in the placebo arm was sampled from a beta distribution during the probabilistic sensitivity analysis, based on the proportion of deaths during the trial period (548/5981). The deaths occurring on each day, as a proportion of the overall 28-day risk, remained the same during sampling.

For peer review only

Table 2: Base case cost parameters

Parameter	Value	SE or proportion	Distribution	Source
TXA administration	*£30.01*	-	-	BNF [35], PSSRU[28], Assumption
Inpatient stays to day-28				
Total length of stay (days)				
TXA	*5.83*	1.46	Gamma	HALT-IT
No TXA	*5.80*	1.47	Gamma	HALT-IT
Days in ICU				
TXA	*0.4*	0.46	Gamma	HALT-IT
No TXA	*0.4*	0.51	Gamma	HALT-IT
Unit cost per day non-ICU	*£338*	-	-	Reference costs [38]
Unit cost per day on ICU	*£1,594*	-	-	Reference costs [38]
Procedures to day-28				
Prob endoscopy - diagnostic				
TXA	*0.8*	4,781/5,953	Beta	HALT-IT
No TXA	*0.79*	4,729/5,978	Beta	HALT-IT
Unit cost	*£665*	-	-	Reference costs [38]
Prob endoscopy - therapeutic				
TXA	*0.43*	2,542/5,953	Beta	HALT-IT
No TXA	*0.44*	2,658/5,978	Beta	HALT-IT
Unit cost	*£777*	-	-	Reference costs [38]
Prob surgical intervention				
TXA	*0.02*	146/5,953	Beta	HALT-IT
No TXA	*0.03*	158/5,978	Beta	HALT-IT
Unit cost	*£1,377*	-	-	Reference costs [38]
Prob radiological - diagnostic				
TXA	*0.29*	1,704/5,953	Beta	HALT-IT
No TXA	*0.29*	1,744/5,978	Beta	HALT-IT
Unit cost	*£4,986*	-	-	Reference costs [38]
Prob radiological - therapeutic				
TXA	*0.01*	74/5,953	Beta	HALT-IT
No TXA	*0.01*	89/5,978	Beta	HALT-IT
Unit cost	*£4,986*	-	-	Reference costs [38]
Transfusions				
Prob blood or red cells				
TXA	*0.67*	3,984/5,951	Beta	HALT-IT
No TXA	*0.67*	4,018/5,978	Beta	HALT-IT
Mean units of blood or red cells				
TXA	*2.80*	0.61	Gamma	HALT-IT
No TXA	*2.90*	0.69	Gamma	HALT-IT
Unit cost	*£132*	-	-	NHS Blood & trans. [39]
Prob fresh frozen plasma				
TXA	*0.15*	910/5,951	Beta	HALT-IT
No TXA	*0.16*	993/5,978	Beta	HALT-IT
Mean units fresh frozen plasma				
TXA	*0.90*	0.61	Gamma	HALT-IT
No TXA	*1.0*	0.66	Gamma	HALT-IT
Unit cost	*£31*	-	-	NHS Blood & trans. [39]
Prob platelets				
TXA	*0.04*	219/5,951	Beta	HALT-IT
No TXA	*0.04*	255/5,978	Beta	HALT-IT
Mean units of platelets				
TXA	*0.20*	0.23	Gamma	HALT-IT
No TXA	*0.20*	0.26	Gamma	HALT-IT
Unit cost	*£192*	-	-	NHS Blood & trans. [39]
Post discharge costs				
Year1	*£4,350*	£2,175†	Gamma	Ramagopalan et al. [40]
Year 2	*£1,980*	£990†	Gamma	Ramagopalan et al. [40]
Year 3	*£1,938*	£969†	Gamma	Ramagopalan et al. **[40]**

†standard errors were assumed to be 50% of the mean value in the base case analysis

For peer review only

For peer review only - http://bmjopen.bmj.com/site/about/guidelines.xhtml

Table 3: Base case deterministic results, including a breakdown of cost components

	No TXA	TXA
Total costs	£18,220	£18,155
TXA costs	£0	£30
Procedures and transfusions	£5,162	£5,112
Monitoring costs	£13,057	£13,013
Life Years	12.08	12.04
QALYs	8.61	8.58
Incremental costs		-£64.30
Incremental QALYs		-0.041
ICER(LY)		£1,576
ICER (QALY)		£2,209

For peer review only

1
2 *Table 4: Deterministic one-way sensitivity analysis results*

	Inc. costs (£)[†]	Inc. LY[†]	Inc. QALY[†]	ICER LY (£)	ICER QALY (£)	INMB[‡] (QALY) (£)
**Base case**	-64	-0.041	-0.029	1,576	2,209	-518
Monitoring costs (full monitoring costs for year 4 onwards)	-106	-0.041	-0.029	2,605	3,652	-476
Monitoring costs not included beyond 3 years	-43	-0.041	-0.029	1,061	1,488	-539
0% discount rate	-78	-0.060	-0.043	1,291	1,828	-775
6% discount rate	-58	-0.032	-0.023	1,799	2,508	-406
Utility post discharge (0.5)	-64	-0.041	-0.020	1,576	3,297	-326
Monitoring costs - inpatient costs excluded in year 1	-55	-0.041	-0.029	1,341	1,880	-527
TXA all-cause mortality risk ratio (0.92)	85	0.097	0.069	875*	1,227*	1304
TXA all-cause mortality risk ratio (1.16)	-231	-0.195	-0.139	1,185	1,662	-2548
Total TXA administration at £0	-94	-0.041	-0.029	2,311	3,240	-488

3 [†]Negative costs, LYs and QALYs indicate that TXA is less costly and less effective than no TXA.
4 [‡] Incremental net monetary benefit (INMB) calculated using a £20,000 willingness to pay for an additional QALY
5 threshold.
6 *In this scenario, the ICERs flip meaning they favour TXA treatment (hence the positive INMB).
7
8

For peer review only - http://bmjopen.bmj.com/site/about/guidelines.xhtml

Figures

Figure 1: Markov Model structure showing both health states within the model.

For peer review only

For peer review only - http://bmjopen.bmj.com/site/about/guidelines.xhtml

Figure 2: Base case cost-effectiveness plane

The dotted line represents the £20,000 willingness to pay threshold; points falling on or below this line are considered cost-effective.
The NE quadrant contains 15% of simulations; SW quadrant 38%; SE quadrant 14%; NW quadrant 33%

For peer review only

For peer review only - http://bmjopen.bmj.com/site/about/guidelines.xhtml

Figure 3: Base case cost-effectiveness acceptability curve

For peer review only

For peer review only - http://bmjopen.bmj.com/site/about/guidelines.xhtml

Statements

Contributorship statement: All authors (NB, AM, IR, HSS, VJ and JW) contributed to the study conception and design. The economic model was developed by NB and JW. The economic analyses were performed by NB, AM, and JW. All authors (NB, AM, IR, HSS, VJ and JW) were involved in the interpretation of the study results. The first draft of the manuscript was written by NB. All authors (NB, AM, IR, HSS, VJ and JW) provided critical review of the manuscript, and have read and approved the final manuscript.

Competing interests: The authors declare that they have no financial or non-financial interests to disclose, and no conflicts of interest to declare.

Funding: This work was supported by the UK National Institute for Health Research Health Technology Assessment Programme (HTA/11/01/04).

Data sharing statement: For the HALT-IT trial data, individual de-identified patient data, including data dictionary, will be made available via our data sharing portal, The Free Bank of Injury and Emergency Research Data (freeBIRD) website indefinitely (https://freebird.lshtm.ac.uk/). The economic model is available from the authors upon request.

Ethics approval: The HALT-IT trial was approved by the UK NRES Committee East of England (reference number 12/EE/0038), and by the national and local research ethics committees in all participating non-UK countries.

For peer review only - http://bmjopen.bmj.com/site/about/guidelines.xhtml

References

1. Hearnshaw, S., et al., *Acute upper gastrointestinal bleeding in the UK: Patient characteristics, diagnoses and outcomes in the 2007 UK audit.* Gut, 2011;60(1327-35.
2. McPherson, S., et al., *Gastrointestinal Haemorrhage: Time to Get Control.* London, UK: National Confidential Enquiry into Patient Outcome and Death, 2015;
3. Crooks, C., T. Card, and J. West, *Reductions in 28-day mortality following hospital admission for upper gastrointestinal hemorrhage.* Gastroenterology, 2011;141(1):62-70.
4. Henderson, R. *Oesophageal Varices*. Gastroenterology 2016 [cited 2020 18 July]; Available from: https://patient.info/doctor/oesophageal-varices#.
5. Hwang, J.H., et al., *The role of endoscopy in the management of acute non-variceal upper GI bleeding.* Gastrointestinal Endoscopy, 2012;75(6):1132-1138.
6. Campbell, H.E., et al., *Costs and quality of life associated with acute upper gastrointestinal bleeding in the UK: cohort analysis of patients in a cluster randomised trial.* BMJ open, 2015;5(4):e007230.
7. Wallis, J.P., A.W. Wells, and C.E. Chapman, *Changing indications for red cell transfusion from 2000 to 2004 in the North of England.* Transfusion Medicine, 2006;16(6):411-417.
8. Villanueva, C., et al., *Transfusion Strategies for Acute Upper Gastrointestinal Bleeding.* New England Journal of Medicine, 2013;368(1):11-21.
9. Dunn, C.J. and K.L. Goa, *Tranexamic acid.* Drugs, 1999;57(6):1005-1032.
10. Vermylen, J., et al., *A double blind study of the effect of tranexamic acid in essential menorrhagia.* Thrombosis and Haemostasis, 1968;20(02):583-587.
11. Srinil, S. and U. Jaisamrarn, *Treatment of idiopathic menorrhagia with tranexamic acid.* JOURNAL-MEDICAL ASSOCIATION OF THAILAND, 2005;88(S1.
12. Wardrop, D., et al., *Antifibrinolytics (lysine analogues) for the prevention of bleeding in patients with haematological disorders.* Cochrane Database of Systematic Reviews, 2013;7):
13. Watterson, C. and N. Beacher, *Preventing perioperative bleeding in patients with inherited bleeding disorders.* Evidence-Based Dentistry, 2017;18(1):28-29.
14. Nutbeam, T., *In adult patients presenting as emergencies with upper gastrointestinal bleeding, does tranexamic acid decrease mortality?* African Journal of Emergency Medicine, 2015;5(2):85-92.
15. The CRASH-3 Trial Collaborators, *Effects of tranexamic acid on death, disability, vascular occlusive events and other morbidities in patients with acute traumatic brain injury (CRASH-3): a randomised, placebo-controlled trial.* The Lancet, 2019;394(10210):1713-1723.
16. Shakur, H., et al., *Effect of early tranexamic acid administration on mortality, hysterectomy, and other morbidities in women with post-partum haemorrhage (WOMAN): an international, randomised, double-blind, placebo-controlled trial.* The Lancet, 2017;389(10084):2105-2116.
17. Olldashi, F., et al., *Effects of tranexamic acid on death, vascular occlusive events, and blood transfusion in trauma patients with significant haemorrhage (CRASH-2): a randomised, placebo-controlled trial.* The Lancet, 2010;376(9734):23-32.
18. Roberts, I., D. Prieto-Merino, and D. Manno, *Mechanism of action of tranexamic acid in bleeding trauma patients: an exploratory analysis of data from the CRASH-2 trial.* Critical Care, 2014;18(6):685.
19. Roberts, I., et al., *Effects of a high-dose 24-h infusion of tranexamic acid on death and thromboembolic events in patients with acute gastrointestinal bleeding (HALT-IT): an international randomised, double-blind, placebo-controlled trial.* The Lancet, 2020;395(10241):1927-1936.
20. Hawkey, G., et al., *Drug treatments in upper gastrointestinal bleeding: value of endoscopic findings as surrogate end points.* Gut, 2001;49(3):372-379.
21. Cormack, F., et al., *Tranexamic acid in upper gastrointestinal haemorrhage.* The Lancet, 1973;301(7814):1207-1208.
22. Bennett, C., et al., *Tranexamic acid for upper gastrointestinal bleeding.* Cochrane Database of Systematic Reviews, 2014;11):
23. Twum-Barimah, E., et al., *Systematic review with meta-analysis: the efficacy of tranexamic acid in upper gastrointestinal bleeding.* Alimentary Pharmacology & Therapeutics, 2020;
24. National Institute for Health and Care Excellence, *Acute upper gastrointestinal bleeding in over 16s: management [CG141].* 2012;
25. Oakland, K., et al., *Diagnosis and management of acute lower gastrointestinal bleeding: guidelines from the British Society of Gastroenterology.* Gut, 2019;68(5):776.
26. Roberts, I., et al., *A high-dose 24-hour tranexamic acid infusion for the treatment of significant gastrointestinal bleeding: HALT-IT RCT.* Health Technol Assess, 2021;25(58):1-86.

For peer review only - http://bmjopen.bmj.com/site/about/guidelines.xhtml

27. National Institute for Health and Care Excellence, *Guide to the methods of technology appraisal.* National Institute for Health and Clinical Excellence (NICE) London, UK, 2018;
28. Jones, K. and A. Burns, *Unit Costs of Health and Social Care 2021*, in *Personal Social Services Research Unit*. 2021, University of Kent: Canterbury.
29. Williams, J., et al., *Cost-effectiveness analysis of tranexamic acid for the treatment of traumatic brain injury, based on the results of the CRASH-3 randomised trial: a decision modelling approach.* BMJ global health, 2020;5(9):
30. Office for National Statistics. *National life tables, UK: 2016 to 2018*. Available from: https://www.ons.gov.uk/peoplepopulationandcommunity/birthsdeathsandmarriages/lifeexpectancies/bulletins/nationallifetablesunitedkingdom/2016to2018.
31. Crooks, C.J., T.R. Card, and J. West, *Excess long-term mortality following non-variceal upper gastrointestinal bleeding: a population-based cohort study.* PLoS medicine, 2013;10(4):e1001437.
32. Jairath, V., et al., *Restrictive versus liberal blood transfusion for acute upper gastrointestinal bleeding (TRIGGER): a pragmatic, open-label, cluster randomised feasibility trial.* Lancet, 2015;386(9989):137-44.
33. Katz, S., et al., *Studies of the illness in the aged, the index of ADL: A standarsized measure of biological and psychological functioning.* JAMA, 1963;185(914-9.
34. Kind, P., et al., *Variations in population health status: results from a United Kingdom national questionnaire survey.* BMJ, 1998;316(
35. Royal Pharmaceutical Society. *British National Formulary*. 2019 14/02/2022]; Available from: https://about.medicinescomplete.com/publication/british-national-formulary/.
36. Guerriero, C., et al., *Cost-Effectiveness Analysis of Administering Tranexamic Acid to Bleeding Trauma Patients Using Evidence from the CRASH-2 Trial.* PLoS One, 2011;6(5):e18987.
37. Li, B., et al., *Tranexamic acid for treatment of women with post-partum haemorrhage in Nigeria and Pakistan: a cost-effectiveness analysis of data from the WOMAN trial.* The Lancet. Global health, 2018;6(2):e222-e228.
38. NHS Improvement. *National schedule of reference costs 2017/18*. Available from: https://webarchive.nationalarchives.gov.uk/20200501111106/https://improvement.nhs.uk/resources/reference-costs/.
39. NHS Blood and Transport. *NHS Blood and Transport Price List 2018/2019*. Available from: https://nhsbtdbe.blob.core.windows.net/umbraco-assets-corp/14810/price_list_bc_nhs_2018-19.pdf.
40. Ramagopalan, S.V., et al., *Costs of gastrointestinal bleeding events in atrial fibrillation: a UK Clinical Practice Research Datalink study.* Future cardiology, 2019;15(5):367-375.
41. Gayet-Ageron, A., et al., *Effect of treatment delay on the effectiveness and safety of antifibrinolytics in acute severe haemorrhage: a meta-analysis of individual patient-level data from 40 138 bleeding patients.* The Lancet, 2018;391(10116):125-132.

For peer review only - http://bmjopen.bmj.com/site/about/guidelines.xhtml

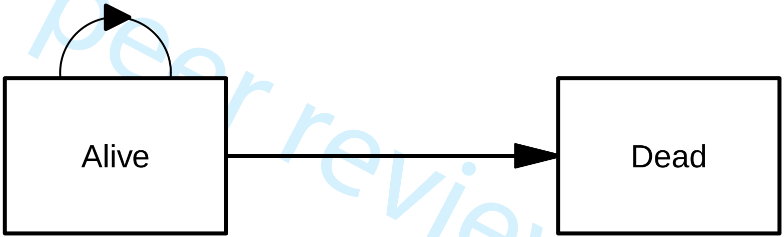

For peer review only - http://bmjopen.bmj.com/site/about/guidelines.xhtml

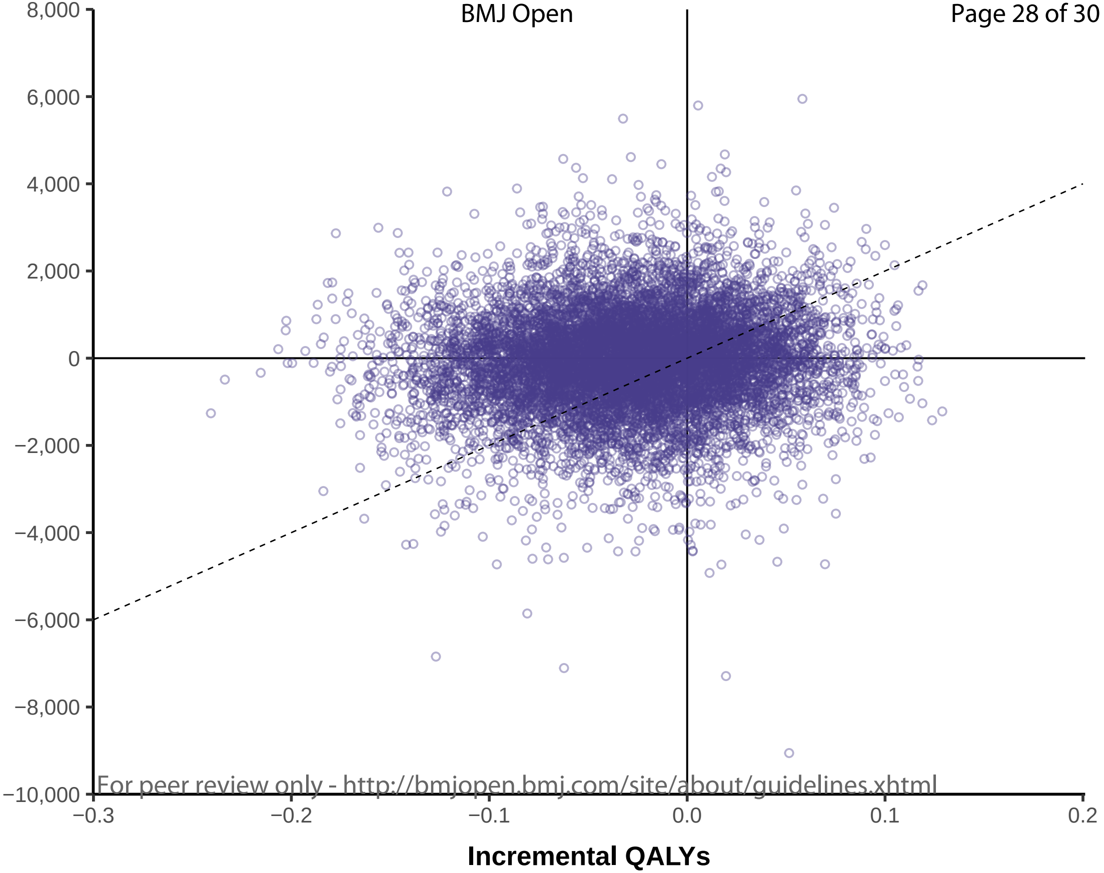

For peer review only - http://bmjopen.bmj.com/site/about/guidelines.xhtml

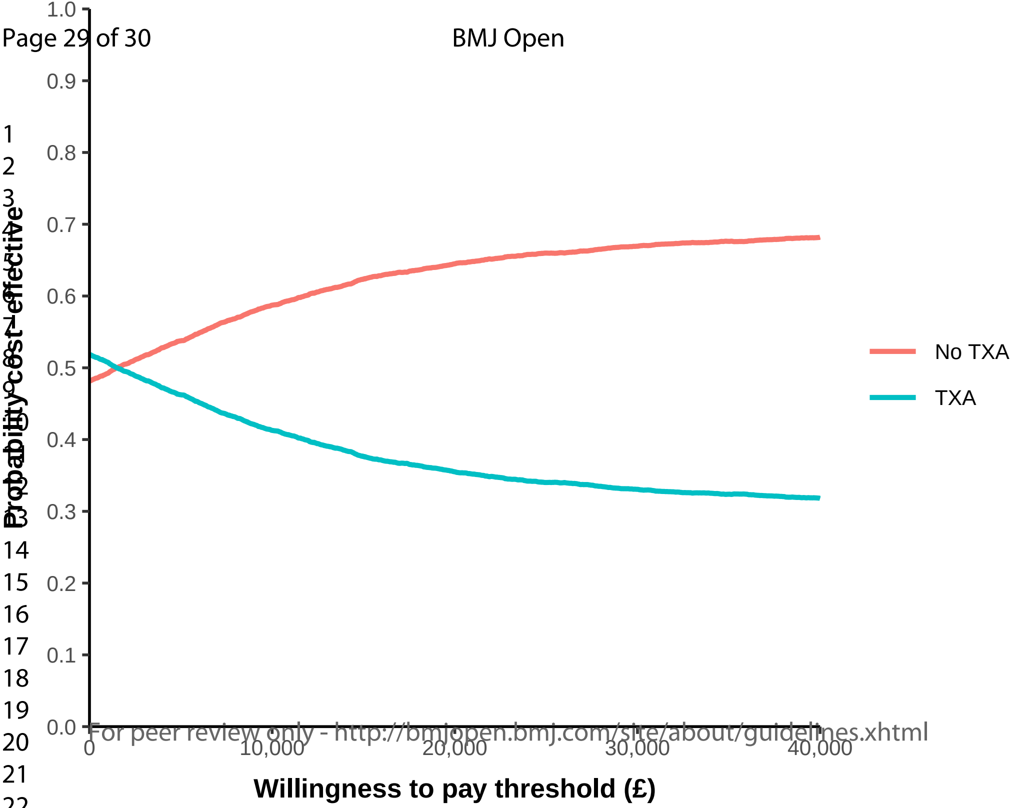

For peer review only - http://bmjopen.bmj.com/site/about/guidelines.xhtml

Table

Table 1| CHEERS checklist—Items to include when reporting economic evaluations of health interventions

Section/item	Item No	Recommendation	Reported on page No/ line No
**Title and abstract**			
Title	1	Identify the study as an economic evaluation or use more specific terms such as "cost-effectiveness analysis", and describe the interventions compared.	P1 L1-3
Abstract	2	Provide a structured summary of objectives, perspective, setting, methods (including study design and inputs), results (including base case and uncertainty analyses), and conclusions.	P3
**Introduction**			
Background and objectives	3	Provide an explicit statement of the broader context for the study.	P6 L13-17
		Present the study question and its relevance for health policy or practice decisions.	P6 L19-27
**Methods**			
Target population and subgroups	4	Describe characteristics of the base case population and subgroups analysed, including why they were chosen.	P7 L3-4, L12-14, P7 L26-27
Setting and location	5	State relevant aspects of the system(s) in which the decision(s) need(s) to be made.	P7 L6
Study perspective	6	Describe the perspective of the study and relate this to the costs being evaluated.	P7 L6
Comparators	7	Describe the interventions or strategies being compared and state why they were chosen.	P7 L3-5
Time horizon	8	State the time horizon(s) over which costs and consequences are being evaluated and say why appropriate.	P7 L5-6
Discount rate	9	Report the choice of discount rate(s) used for costs and outcomes and say why appropriate.	P7 L8-9
Choice of health outcomes	10	Describe what outcomes were used as the measure(s) of benefit in the evaluation and their relevance for the type of analysis performed.	P7 L7-8
Measurement of effectiveness	11a	*Single study-based estimates:* Describe fully the design features of the single effectiveness study and why the single study was a sufficient source of clinical effectiveness data.	P8 L12-18
	11b	*Synthesis-based estimates:* Describe fully the methods used for identification of included studies and synthesis of clinical effectiveness data.	
Measurement and valuation of preference based outcomes	12	If applicable, describe the population and methods used to elicit preferences for outcomes.	P8 L22-25
Estimating resources and costs	13a	*Single study-based economic evaluation:* Describe approaches used to estimate resource use associated with the alternative interventions. Describe primary or secondary research methods for valuing each resource item in terms of its unit cost. Describe any adjustments made to approximate to opportunity costs.	
	13b	*Model-based economic evaluation:* Describe approaches and data sources used to estimate resource use associated with model health states. Describe primary or secondary research methods for valuing each resource item in terms of its unit cost. Describe any adjustments made to approximate to opportunity costs.	P9 L6 - P10 L23
Currency, price date, and conversion	14	Report the dates of the estimated resource quantities and unit costs. Describe methods for adjusting estimated unit costs to the year of reported costs if necessary. Describe methods for converting costs into a common currency base and the exchange rate.	L7 L6-7
Choice of model	15	Describe and give reasons for the specific type of decision-analytical model used. Providing a figure to show model structure is strongly recommended.	P7 L17-19 Figure 1
Assumptions	16	Describe all structural or other assumptions underpinning the decision-analytical model.	P7 L26-27, P9 L10-15, P10 L18-23 (in methods)
Analytical methods	17	Describe all analytical methods supporting the evaluation. This could include methods for dealing with skewed, missing, or censored data; extrapolation methods; methods for pooling data; approaches to validate or make adjustments (such as half cycle corrections) to a model; and methods for handling population heterogeneity and uncertainty.	P8 L12-19 P8 L26-31 P10 L26 - P11 L11
**Results**			
Study parameters	18	Report the values, ranges, references, and, if used, probability distributions for all parameters. Report reasons or sources for distributions used to represent uncertainty where appropriate. Providing a table to show the input values is strongly recommended.	Table 1, Table 2 P10 L26 - P11 L11
Incremental costs and outcomes	19	For each intervention, report mean values for the main categories of estimated costs and outcomes of interest, as well as mean differences between the comparator groups. If applicable, report incremental cost-effectiveness ratios.	P12 L3-8, Table 3
Characterising uncertainty	20a	*Single study-based economic evaluation:* Describe the effects of sampling uncertainty for the estimated incremental cost and incremental effectiveness parameters, together with the impact of methodological assumptions (such as discount rate, study perspective).	

BMJ: first published as 10.1136/bmj.f1049 on 25 March 2013. Downloaded from http://www.bmj.com/ on 21 December 2021 by guest. Protected by copyright.

No commercial reuse: See rights and reprints http://www.bmj.com/permissions Subscribe: http://www.bmj.com/subscribe

BMJ 2013;346:f1049 doi: 10.1136/bmj.f1049 (Published 25 March 2013)

(continued)

Section/item	Item No	Recommendation	Reported on page No/ line No
	20b	*Model-based economic evaluation:* Describe the effects on the results of uncertainty for all input parameters, and uncertainty related to the structure of the model and assumptions.	Figure 2, Figure 3, Table 4
Characterising heterogeneity	21	If applicable, report differences in costs, outcomes, or cost-effectiveness that can be explained by variations between subgroups of patients with different baseline characteristics or other observed variability in effects that are not reducible by more information.	N/A
**Discussion**			
Study findings, limitations, generalisability, and current knowledge	22	Summarise key study findings and describe how they support the conclusions reached. Discuss limitations and the generalisability of the findings and how the findings fit with current knowledge.	P14-15
**Other**			
Source of funding	23	Describe how the study was funded and the role of the funder in the identification, design, conduct, and reporting of the analysis. Describe other non-monetary sources of support.	P23 L10-11
Conflicts of interest	24	Describe any potential for conflict of interest of study contributors in accordance with journal policy. In the absence of a journal policy, we recommend authors comply with International Committee of Medical Journal Editors recommendations.	P23 L7-8

For consistency, the CHEERS statement checklist format is based on the format of the CONSORT statement checklist

BMJ: first published as 10.1136/bmj.f1049 on 25 March 2013. Downloaded from http://www.bmj.com/ on 21 December 2021 by guest. Protected by copyright.

No commercial reuse: See rights and reprints http://www.bmj.com/permissions Subscribe: http://www.bmj.com/subscribe

BMJ Open

An economic evaluation of tranexamic acid for the treatment of acute gastrointestinal bleeding: A cost-effectiveness analysis using data from the HALT-IT randomised controlled trial

Journal:	*BMJ Open*
Manuscript ID	bmjopen-2021-060505.R2
Article Type:	Original research
Date Submitted by the Author:	01-Apr-2022
Complete List of Authors:	Bazeer, Nuha; London School of Hygiene & Tropical Medicine, London, UK and London School of Economics and Political Science, London, UK<br>Miners, Alec; London School of Hygiene & Tropical Medicine, Department of Health Services Research and Policy<br>Roberts, Ian; London School of Hygiene & Tropical Medicine, Clinical Trials Unit<br>Shakur-Still, Haleema; London School of Hygiene & Tropical Medicine, Clinical Trials Unit<br>Jairath, Vipul; Western University, Department of Medicine, Division of Gastroenterology<br>Williams, Jack; London School of Hygiene & Tropical Medicine, Department of Health Services Research and Policy
**Primary Subject Heading**:	Health economics
Secondary Subject Heading:	Gastroenterology and hepatology, Haematology (incl blood transfusion)
Keywords:	GASTROENTEROLOGY, Bleeding disorders & coagulopathies < HAEMATOLOGY, HEALTH ECONOMICS

SCHOLARONE™ Manuscripts

I, the Submitting Author has the right to grant and does grant on behalf of all authors of the Work (as defined in the below author licence), an exclusive licence and/or a non-exclusive licence for contributions from authors who are: i) UK Crown employees; ii) where BMJ has agreed a CC-BY licence shall apply, and/or iii) in accordance with the terms applicable for US Federal Government officers or employees acting as part of their official duties; on a worldwide, perpetual, irrevocable, royalty-free basis to BMJ Publishing Group Ltd ("BMJ") its licensees and where the relevant Journal is co-owned by BMJ to the co-owners of the Journal, to publish the Work in this journal and any other BMJ products and to exploit all rights, as set out in our licence.

The Submitting Author accepts and understands that any supply made under these terms is made by BMJ to the Submitting Author unless you are acting as an employee on behalf of your employer or a postgraduate student of an affiliated institution which is paying any applicable article publishing charge ("APC") for Open Access articles. Where the Submitting Author wishes to make the Work available on an Open Access basis (and intends to pay the relevant APC), the terms of reuse of such Open Access shall be governed by a Creative Commons licence – details of these licences and which Creative Commons licence will apply to this Work are set out in our licence referred to above.

Other than as permitted in any relevant BMJ Author's Self Archiving Policies, I confirm this Work has not been accepted for publication elsewhere, is not being considered for publication elsewhere and does not duplicate material already published. I confirm all authors consent to publication of this Work and authorise the granting of this licence.

For peer review only

An economic evaluation of tranexamic acid for the treatment of acute gastrointestinal bleeding: A cost-effectiveness analysis using data from the HALT-IT randomised controlled trial

Authors:

Nuha Bazeer[1] (0000-0002-4851-8402)

Alec Miners[2] (0000-0003-1850-1463)

Ian Roberts[3] (0000-0003-1596-6054)

Haleema Shakur-Still[3] (0000-0002-6511-109X)

Vipul Jairath[4] (0000-0002-1092-0033)

Jack Williams[2] (0000-0002-1331-387X)

1 London School of Hygiene & Tropical Medicine, London, UK and London School of Economics and Political Science, London, UK

2 Department of Health Services Research and Policy, London School of Hygiene & Tropical Medicine, London, UK

3 Clinical Trials Unit, London School of Hygiene & Tropical Medicine, London, UK

4 Department of Medicine, Division of Gastroenterology, Western University, London, Canada

For peer review only

For peer review only - http://bmjopen.bmj.com/site/about/guidelines.xhtml

Corresponding author:
Jack Williams
London School of Hygiene & Tropical Medicine
Room 142, 15-17 Tavistock Place, London, WC1H 9SH
Jack.Williams@lshtm.ac.uk

For peer review only

Abstract

Objective: To perform an economic evaluation of tranexamic acid (TXA) versus no TXA, in additional to current clinical practice, for acute gastrointestinal bleeding, using the results of the HALT-IT trial, a large randomised controlled trial which included 11,937 patients.

Design: A cost-effectiveness modelling analysis, performed over a lifetime time horizon.

Setting: The analysis was performed from a UK health service perspective

Participants: The model includes adults with acute gastrointestinal bleeding.

Outcomes measures: The model reports costs in Great British Pounds (GBP) in 2021 and outcomes as life years (LYs) and quality-adjusted life years (QALYs). Cost-effectiveness was evaluated using incremental cost-effectiveness ratios (ICERs), reported as the cost per QALY gained.

Methods: A Markov model was developed to calculate the overall costs and health outcomes of TXA administration versus no TXA. The model used data of the treatment effectiveness from the HALT-IT trial, which showed that TXA administration for acute gastrointestinal bleeding did not reduce all-cause mortality (RR 1.03, 95% CI 0.92; 1.16) compared to no TXA. Data on health-related quality of life, costs, and long-term mortality risks were derived from the literature. Costs and effects are discounted at 3.5% per annum.

Results: TXA was associated with marginally fewer LYs and QALYs, and lower costs, than treatment without TXA. The ICER associated with no TXA was £1,576 per LY gained and £2,209 per QALY gained. No TXA was 64% likely to be cost-effective at a £20,000 willingness to pay threshold, whilst TXA was 36% likely to be cost-effective.

Conclusion: Though inexpensive, TXA administration for patients with acute gastrointestinal bleeding is unlikely to be cost-effective.

For peer review only

For peer review only - http://bmjopen.bmj.com/site/about/guidelines.xhtml

Strengths and limitations of this study

- This is the first economic evaluation of tranexamic acid for the treatment of acute gastrointestinal bleeding
- Data for the treatment effect comes from the HALT-IT trial, which included 11,937 patients receiving either tranexamic acid or placebo.
- The cost-effectiveness model extrapolates the impact of tranexamic acid treatment over a lifetime time horizon, as recommended by the National Institute for Health and Care Excellence, for UK based economic evaluations
- The HALT-IT trial data only reports outcomes up to 28 days following the onset of acute gastrointestinal bleeding, and therefore the model only accounts for treatment effects observed within this 28 day trial period.

For peer review only

For peer review only - http://bmjopen.bmj.com/site/about/guidelines.xhtml

Introduction

Acute gastrointestinal bleeding (AGIB) refers to bleeding arising from the upper or lower gastrointestinal tract. Acute upper gastrointestinal bleeding (AUGIB) occurs more commonly than acute lower gastrointestinal bleeding (ALGIB) and has a higher case fatality rate of up to 10% [1]. AUGIB is a common medical emergency and results in approximately 85,000 cases each year within the United Kingdom, with around 4,000 AUGIB related deaths annually [2]. Thus, AUGIB presents a significant health concern with an estimated incidence of 133 cases per 100,000 population, accounting for 8% of all acute hospital admissions [2]. The leading causes of AUGIB have been recorded as peptic ulcers and esophagogastric varices [1, 3]. While peptic ulcers are often associated with long-term use of non-steroid anti-inflammatory drugs (NSAIDs), esophagogastric varices are typically complications of liver cirrhosis [4]. Comparisons of national AUGIB audits in the United Kingdom carried out in 1994 and 2007 have shown improved outcomes amongst patients, likely through advancements in endoscopic and some pharmacological therapies [1]. However, over the past two decades, there have been no significant changes to mortality associated with AUGIB, and mortality amongst older patients with other comorbidities remains high at 10% [2, 5]. Within the UK, annual in-hospital costs for all AUGIB cases have been estimated as £155.5 million, of which £12.6 million accounts for blood product transfusions (BPT) alone [6]. As the second most common medical reason for transfusion, GI bleeding accounts for 14% of all BPT within the UK, with inappropriate use recorded in 20% of cases [7, 8].

Tranexamic acid (TXA) is an anti-fibrinolytic agent which reduces fibrinolysis and inhibits the breakdown of blood clots [9]. It's used to treat women with heavy menstrual bleeding (menorrhagia) and people with haemophilia during dental extractions [10-13]. The efficacy of TXA has also been demonstrated in reducing blood loss during surgical operation trials [9]. It is widely available, relatively inexpensive, and can be administered through oral or intravenous formulations, without specialist monitoring [14]. Large, multi-centre, randomised controlled trials (RCTs) such as the WOMAN and CRASH-2 and -3 trials have shown TXA to significantly reduce bleeding mortality amongst patients at risk of postpartum haemorrhage and bleeding trauma patients respectively [15-17]. However, the timing of TXA administration is of particular importance, with no evidence of a benefit upon mortality when administered more than three hours after injury in both WOMAN and CRASH-2 and -3 trials. [16, 18]. Unlike postpartum haemorrhage or brain injury however, the onset of a gastrointestinal bleed may be less discernible, with presentation often delayed due to symptoms only becoming

For peer review only - http://bmjopen.bmj.com/site/about/guidelines.xhtml

visible when most severe. This was observed within the HALT-IT trial, with over 80% of patients presenting more than three hours after the onset of bleeding [19].

Previously conducted smaller RCTs in the UK have found no evidence of a difference in mortality, rebleeding, and the requirement of surgery [20, 21]. However, a 2014 Cochrane systematic review and meta-analysis of RCTs comparing TXA with placebo for AUGIB estimated large reductions in all-cause mortality, with a pooled risk ratio (RR) of 0.6 (95% CI, 0.42–0.87) across 8 studies with 1701 participants [22]. However, the mortality benefit of TXA was uncertain when excluding trials with a high risk of bias. For this reason, the authors concluded that tranexamic acid cannot be recommended for routine clinical practice. An updated systematic review and meta-analysis found similar findings, with a risk ratio of 0.59 (95% CI 0.43-0.82), and also concluded that high-quality RCTs were needed [23].

TXA is not currently recommended within UK guidelines for AUGIB or ALGIB [24]. National Institute for Health and Care Excellence (NICE) guidelines for AUGIB refer to limited evidence of an effect of TXA on mortality, but do not include recommendations for TXA in the absence of UK marketing authorisation for this indication. Similarly, guidelines from the British Society of Gastroenterology state that the mortality benefit of TXA is uncertain when excluding trials with a low risk of bias, suggesting recommendations are made following the HALT-IT trial [25].

The HALT-IT trial was a large multinational RCT that compared TXA versus placebo, in addition to current practice, in 11,937 people with AGIB [19]. The trial protocol has also been published [26]. The trial did not find evidence that TXA significantly reduces any of the key outcome measures, such as death due to bleeding within 5 days of randomisation (RR 0.99; 95% CI 0.82; 1.18) or all-cause mortality (RR 1.03 95% CI 0.92; 1.16). This means by default, even if relatively inexpensive, TXA is unlikely to represent value for money in this indication. However, there is still a need to perform an economic evaluation based on the HALT-IT results, in order to establish the level of uncertainty about this conclusion and to assess the extent to which only including the results from the systematic review would have impacted the cost-effectiveness estimates. The full results of the HALT-IT trial, including the economic results, are available in a separate National Institute of Health Research report [27].

For peer review only - http://bmjopen.bmj.com/site/about/guidelines.xhtml

Methods

Analysis

The base case economic analysis assessed the cost-effectiveness of treating patients identified as sustaining an AGIB with TXA versus no TXA, in addition to current practice, using estimates of treatment effect from the HALT-IT trial. In line with NICE guidelines for health technology appraisals, A cost-effectiveness model was developed in Microsoft Excel and was analysed over a lifetime time horizon, from a UK NHS health service perspective [28]. All costs are reported in 2021 Great British Pounds, with costs inflated using an NHS cost inflation index [29]. Health outcomes were assessed as life years (LYs), and quality-adjusted life years (QALYs). All future costs and health outcomes were discounted at 3.5% per annum, as per NICE guidance [28]. We report the results as incremental cost-effectiveness ratios (ICERs) by diving the incremental costs by the incremental outcomes. We also report results using incremental net monetary benefits (INMBs), by converting incremental outcomes in monetary values based on the willingness to pay threshold, and then subtracting the incremental costs. This means that positive INMB represents a cost-effective scenario, whilst a negative INMB does not. A willingness to pay threshold of £20,000 per QALY has been used to determine cost-effectiveness, in line with NICEs Technology Appraisals recommendations [28].

The HALT-IT trial included a number of sub-group analyses, but as no treatment effects were observed amongst subgroups, they were not considered in the model. Whilst the trial consisted mostly of patients with AUGIB (89%), the model population includes both AUGIB and ALGIB, as per the overall trial population.

Model Structure

An existing Markov model of TXA following traumatic brain injury was adapted to estimate the cost-effectiveness of TXA for patients with AGIB [30]. The model consists of two health states *alive* and *dead*; as shown in Figure 1. People with AGIB enter the model in the *alive* health state. They progress to the *dead* health state over the initial 28-day period (the HALT-IT trial period) using daily data taken directly from the trial on all-cause deaths from the placebo arm of HALT-IT; this includes data for all UK and non-UK participants. For the remaining 337 days of the first year, the daily probability of death in the no-TXA arm was estimated using UK general population age-related mortality statistics [31], adjusted using a standardised mortality ratio (SMR) to reflect the long-term risk of excess death associated with surviving an AGIB [32]. In the base case, this SMR is

For peer review only

applied for the entire time horizon. After this initial year period, the model uses an annual cycle length, and applies a probability of death based on UK all-cause mortality data, combined with the SMR. In line with the HALT-IT trial, the mean age of people entering the model was assumed to be 58.1 years (Table 1).

The SMR associated with the risk of death following an AGIB compared to that of the general population was derived from a study by Crooks et al (2013) [32]. It linked longitudinal data from the UKs Hospital Episodes Statistics dataset, General Practice Research Database and Office of National Statistics death register for people with non-variceal bleeds between 1997 and 2010. Controls were matched by age, sex, practice, and year. The study reported the risk of death was highest in the first year following an AGIB (SMR 5.21 95% CI 4.25; 6.38), and remained elevated from year 2 onwards (SMR 1.74 95% CI 1.42; 2.13).

Treatment Effect

The HALT-IT trial results for the primary outcome measure, death due do bleeding within 5 days from randomisation, was similar in both treatment arms (RR 0.99; 95% CI 0.82; 1.18), although the mean estimate slightly favoured TXA.[19] However, as the risks of venous thromboembolic events (RR 1.85; 95% CI 1.15; 2.98), death from malignancy (RR 1.63; 95% CI 1.10; 2.42), and seizure (RR 1.73; 95% CI 1.03; 2.93) were higher in the TXA- than placebo-group, the base case RR of all-cause mortality was used to estimate the overall effect of TXA treatment on survival (RR 1.03 95% CI 0.92; 1.16), and is shown in Table 1. This RR is based on all trial participants and is not UK specific since the subgroup analysis did not reveal any differences in treatment effect by country income level.

Utilities

Utility data were not collected as part of HALT-IT, therefore values were sourced from the existing literature. Only one directly relevant study could be identified – the relatively recent TRIGGER RCT [33]. The EQ-5D-3L questionnaire was administered at discharge or day-28, with an estimated utility of 0.735 (95% CI 0.70; 0.77) for those surviving an AGIB (Table 1) [6]. In line with previous economic evaluations of TXA, this single utility value of 0.735 was applied to all people who were alive at day-28, both during the initial 28-day period and at all times thereafter until death, in both treatment arms [30]. Note that functional status was measured in HALT-IT using the Katz Index of Independence in Activities of Daily Living [34], with the scores similar in both arms (score of 5.5 for both).

For peer review only - http://bmjopen.bmj.com/site/about/guidelines.xhtml

Since the TRIGGER RCT population has a slightly lower level of co-morbidities and complications amongst participants compared to the HALT-IT population, we considered the impact of a much lower utility value (0.5) for all surviving patients following AGIB.

An age adjustment was also applied to the utility value each cycle using the decrements reported by Kind et al., so that overall values declined with age [35].

Costs

Treatment costs

The resource use associated with providing TXA treatment were derived from the HALT-IT trial and by making a number of assumptions. The total TXA dose (4g) included a loading- and maintenance-dose, for which the total cost was derived from the British National Formulary (£12 per patient) [36]. The cost of equipment for treatment was assumed to include a needle (£0.05), syringe (£0.07), sodium chloride infusion bag (£0.59) and isotonic IV solution (£2.96) [36]. To account for the administration time of TXA, hourly staff costs were taken from the Personal Social Services Research Unit for 2021 [29]. The model assumed that a Band 5 nurse would take 21 minutes to administer TXA (£14.35), following the treatment administration and expert guidance used within the economic evaluations of the CRASH [30, 37] and WOMAN trials [38]. This led to a total TXA treatment and administration cost of £30.01 per person (Table 2).

Inpatient stay costs

Information from HALT-IT was used to derive the costs of inpatient stays during the initial 28 day trial period. The total mean lengths of stay recorded by people receiving TXA and placebo were 5.83 and 5.80 days respectively (Table 2). In both treatment arms, 0.4 of these days were spent in intensive care units (ICU). These lengths of stay were multiplied by NHS Reference Costs for non-elective stays to derive mean hospital costs for TXA and no TXA of £2,471 and £2,462 per person respectively [39].

Procedures and Transfusions

While in hospital during the 28-day trial period, participants underwent a number of procedures, including endoscopy, surgery and radiological interventions (Table 2). The cost of these procedures were calculated for each trial arm using the probability of occurrence from the HALT-IT trial multiplied by the NHS

For peer review only - http://bmjopen.bmj.com/site/about/guidelines.xhtml

Reference Costs [39]. The mean per person procedural costs for the TXA and no TXA treatment options were £2,389 and £2,437 respectively.

People also received transfusions of blood / red blood cells, fresh frozen plasma and platelets whilst in hospital (Table 2). Information from HALT-IT on the frequency and the mean number of units for each transfusion type were combined with unit costs from the NHS Blood and Transfusions price list to generate overall costs [40]. The mean per person transfusion costs for the TXA and no TXA treatment options were £253 and £264 respectively.

Whilst the model did not explicitly account for adverse events during the trial period, the costs of these adverse events are included in the hospital length of stay, procedures and transfusions that occur during the hospital stay.

Post discharge costs

The HALT-IT and TRIGGER trials observed participants for a maximum of 28-days, therefore longer-term care costs were sourced from the literature. A specific study related to AGIB could not be identified. However, a recent UK study by Ramagopalan et al. quantified the costs of gastrointestinal bleeding among people with nonvalvular atrial fibrillation (NVAF), who were and were not experiencing a bleed, and used a difference in difference approach to calculate the costs attributable to a gastrointestinal bleed (cases, n=7,753 and controls, n=7,753) [41].

The study used NHS reference costs and calculated the annual costs of caring for people with a bleed to be £4,350, £1,980 and £1,938 in the first second- and third-years post-bleed (Table 2). In the absence of other information, the reported year three costs were inputted into the model for years four onwards, although they were assumed to be one third the original amount. The standard errors that the costing study reports could not be easily incorporated into our analysis (the statistical approach used allowed costs to be negative), therefore they were assumed to be 50% of the mean value in all instances, which is thought to reflect a relatively large degree of uncertainty around the mean estimates. The importance of these assumptions was assessed in the sensitivity analysis.

Sensitivity Analysis

Various one-way deterministic sensitivity analyses were undertaken to assess the robustness of the results to alternative assumptions. In line with NICE guidelines, one-way DSA was conducted on the discount rate for

For peer review only - http://bmjopen.bmj.com/site/about/guidelines.xhtml

both costs and health outcomes, using values of 0% and 6%. It was also conducted on a range of scenarios considering the extrapolation of the monitoring costs, health-related quality of life for AGIB survivors, and TXA treatment effect.

To simultaneously investigate the effect of parameter uncertainty, probabilistic sensitivity analysis (PSA) was also undertaken using 10,000 Monte Carlo simulations; the parameter distributions are specified in Table 1 and Table 2. The probabilistic results are presented on a cost-effectiveness plane, and as a cost-effectiveness acceptability curve (CEAC). Parameters that were excluded from the PSA are not assigned a distribution type in the tables.

A scenario analysis was performed to consider the economic results that would have been generated prior to the availability of the results of TXA from the HALT-IT trial. This analysis was performed primarily to demonstrate the differences in decision making when using low or moderate quality evidence to populate the economic model, particularly from trials with a high risk of bias. In this analysis the 28-day mortality risk was 0.084 (71/850) in the control group, whilst the TXA all-cause mortality RR was 0.60 (95% CI: 0.42-0.87), as per the Cochrane analysis [22].

Patient and Public Involvement

Patient and public input was used to inform the HALT-IT trial procedures, including study information sheets and consent forms [27]. Patients stated that the research should account for quality of life issues in additional to the treatment efficacy and safety, which this economic analysis does. There was no patient or public involvement specifically in the design of the economic model.

For peer review only - http://bmjopen.bmj.com/site/about/guidelines.xhtml

Results

Base Case Analysis

The base case results are shown in Table 3. In both the deterministic and probabilistic analyses TXA was associated with marginally fewer LYs and QALYs, and marginally lower costs, than treatment without TXA. The deterministic QALYs and costs associated with TXA and no-TXA were 8.58 and 8.61, and £18,155 and £18,220 respectively. This meant that providing TXA was £64.30 less costly, and resulted in 0.041 fewer QALYs, per person treated.

Since TXA was less costly and less effective in the base case analysis compared to placebo, the ICERs can be reported as £1,576 saved for each LY lost, and £2,209 saved for each QALY lost. Since we value a QALY at £20,000 using the NICE willingness to pay threshold, this does not represent a cost-effective option, because the value of the health outcomes foregone are higher than the cost savings which occur. Alternatively the results can be expressed as no-TXA versus TXA, with no-TXA costing £1,576 per LY gained and £2,209 per QALY gained, meaning no-TXA is preferred at the NICE willingness to pay threshold. Either way, providing TXA is not cost-effective. The deterministic and mean probabilistic results are very similar.

The lower mean health outcomes for TXA are driven by the fact the mean risk ratio for all-cause mortality (i.e. the relative treatment effect) slightly favours the no-TXA treatment arm (RR 1.03). The higher incremental costs associated with no-TXA administration are largely driven by the fact people who did not receive TXA live for marginally longer than those who received TXA, thus they incur slightly higher post discharge costs, and marginally higher costs for procedures and transfusions, exceeding the additional low cost of TXA administration.

Probabilistic Sensitivity Analysis

The cost-effectiveness plane shows that the PSA simulations are clustered around the origin and distributed across all four cost-effectiveness quadrants (Figure 2). This is because the point estimate for the risk ratio of all-cause mortality is only slightly above one, and is included within its confidence interval (RR 1.03 95% CI 0.92; 1.16). Therefore, there is little to choose between the two treatment options in terms of outcomes. The same is also true of the relatively low cost of TXA administration (£30.01 per person), meaning that the majority of the costs incurred are the hospital costs (hospital stay, procedures and transfusions) and post-discharge costs.

For peer review only

The CEAC (Figure 3) shows that at any willingness to pay for an additional QALY above £2,000, no-TXA is likely to be the more cost-effective option. At the NICE specified willingness to pay threshold levels of £20,000 and £30,000 per additional QALY, TXA has a 36% and 33% probability of being cost-effective, respectively. Note that the base case CEAC does not indicate that there is a 0% probability TXA is cost-effective, irrespective of the willingness to pay value. TXA treatment produced more QALYs at lower cost (i.e. it was the 'dominant' treatment option) in 15% of simulations. Rerunning the PSA assuming the post discharge costs had a standard error of 20%, instead of 50%, of their mean value, had a negligible impact on the probability of TXA being cost-effective.

Deterministic Sensitivity Analysis

Various deterministic one-way sensitivity analyses are shown Table 4. In all but one instance the results show that TXA is not cost-effective, and the ICER was very robust to alternative model parameter and structural assumptions. The exception is when the risk ratio of all-cause mortality is reduced to 0.92 (the lower bound of the 95% CI). In this instance, TXA produces marginally better health outcomes at slightly higher costs than no-TXA, producing an ICER of £1,227 per QALY gained if TXA is used instead of no TXA. Note that even when the cost of TXA and its administration was set to £0 instead of the base case value of £30.01, it is not cost-effective.

Meta-analysis Scenario Analysis

When parameterising the model with the mortality risk and all-cause mortality treatment effect reported from a meta-analysis containing multiple studies at risk of bias, prior to the availability of the HALT-IT trial, TXA becomes highly cost-effective, with an ICER of £1,040 per LY gained and £1,459 per QALY gained. In the probabilistic analysis, TXA was 99.5% likely to be cost-effective at the £20,000 per QALY threshold.

For peer review only - http://bmjopen.bmj.com/site/about/guidelines.xhtml

Discussion

The results from the base case analysis suggest that the costs and outcomes of treating people with AGIB with and without TXA, as enrolled and treated in the HALT-IT trial, are very similar. Despite being a relatively inexpensive intervention, TXA is unlikely to be a cost-effective use of NHS resources [19], and no-TXA is likely to be the cost-effective option. These results are in line with our prior expectations given the additional, albeit modest, cost of providing TXA, which did not reduce mortality. However, the base case probabilistic analysis showed that there is a 36% chance TXA is cost-effective at £20,000 per QALY thresholds, since there was uncertainty around the all-cause mortality treatment effect, with the risk ratio confidence interval ranging from 0.92 to 1.16.

As with all decision models, our analysis includes a number of parameter and structural assumptions. Whilst the analysis calculated the hospital costs associated with the hospital stay and procedures performed, the model did not explicitly include the long-term impacts of adverse events. Instead, the data used to extrapolate the costs and effects following the 28-day trial period were assumed to include AGIB patients with similar co-morbidities and complications following their GI bleed.

However, extensive sensitivity analysis showed that the parameter values for the costs and utilities following AGIB were generally unimportant in terms of changing the conclusion that TXA is unlikely to be cost-effective in this indication. The only sensitivity analysis in which TXA was cost-effective was when it was assumed to reduce mortality. Given that TXA resulted in marginally lower costs than no TXA (due to marginally lower hospital costs during the trial period), it would be cost-effective if it resulted in any all-cause mortality benefit. Given this, if future research identified a modest treatment effect for a subgroup of AGIB patients, then TXA would likely be cost-effective in this group.

To the best of our knowledge, no other cost-effectiveness analyses of TXA for people with AGIB have been reported. However, economic evaluations based on the series of CRASH RCTs and WOMAN RCT have concluded TXA is highly cost-effective following trauma injury and post-partum haemorrhage [15, 16, 30, 38]. The dose of TXA used in the HALT-IT trial is higher than that used in the previous studies, but this increased cost is negligible. The reason for these differences in the cost-effectiveness results is driven by the mortality benefit associated with TXA in other indications, which was not observed the HALT-IT trial population, or in any sub-group analyses. Of considerable importance and highlighted within the WOMAN and CRASH-3 studies was the time to treatment, with earlier treatment (within 3 hours of onset of bleeding) associated with improved

For peer review only - http://bmjopen.bmj.com/site/about/guidelines.xhtml

outcomes [42]. In AGIB patients, it is often difficult to identify the onset of bleeding, and presentation is often delayed. In the HALT-IT trial, 80% of participants received treatment more than 3 hours after the suspected onset of bleeding. Furthermore, patients with AGIB are older, with more comorbidities [19].

One of the main strengths of this analysis is that it is the first economic evaluation of TXA for people with AGIB based on the results of a large RCT. The results from HALT-IT are in contrast to previous meta-analyses of small trials, in which TXA was estimated to result in a large reduction in all-cause mortality in people with AUGIB, when including trials at risk of bias [22, 23]. A scenario analysis using these meta-analysis results found that, had an economic evaluation using the results of this meta-analysis been performed prior to the HALT-IT trial, the results would have suggested TXA was almost certainly cost-effective, with little uncertainty, and indicated little value in performing additional research. With hindsight, we believe the authors of these analyses were correct to conclude that their findings were insufficient to recommend the routine use of TXA in clinical practice, since this would have potentially led to poorer outcomes at a health systems level and potentially impacted on HALT-IT recruitment. For future health economic analyses, we suggest that researchers should be cautious in their use of meta-analyses that include trials considered susceptible to error or bias, which could subsequently result in misleading policy recommendations.

In carrying out an economic evaluation with robust sensitivity analysis, our findings emphasise the importance of large RCTs designed to ascertain the effectiveness of an intervention, and the importance of an economic analysis using high quality RCT data, to assess the cost-effectiveness of low-cost interventions.

Conclusion

Building on the evidence provided from the largest RCT in patients with AGIB, the results of this analysis have shown TXA administration for patients with AGIB is, on balance, unlikely to be cost-effective at UK WTP thresholds, and should not be recommended based on the current economic evidence. However, there remains uncertainty around this conclusion, with TXA being cost-effective in approximately one-third of simulations. This is because TXA would be cost-effective if it resulted in any mortality benefit, and the confidence interval for the all-cause mortality treatment effect observed in the HALT-IT trial (0.92-1.16), does not rule out such a finding. These results also highlight that if a modest treatment effect for a subset of patients can be identified in the future, then TXA has considerable scope to be cost-effective for this group.

For peer review only - http://bmjopen.bmj.com/site/about/guidelines.xhtml

Tables

Table 1: Base case model input parameters

Parameter	Value	95% CIs	Distribution	Source
Age at model start	*58.1*	-	-	HALT-IT
SMR year 1	*5.21*	4.25; 6.38	Log normal	Crooks et al [32]
SMR year 2	*1.74*	1.42; 2.13	Log normal	Crooks et al [32]
RR of all-cause mortality	*1.03*	0.92; 1.16	Log normal	HALT-IT
28-day probability of death†	*0.092*	0.084; 0.099	Beta	HALT-IT
Utility AGIB survivor	*0.735*	0.70; 0.77	Beta	Campbell et al [6]
Utility decrements by age (years)				Kind et al [35]
55-64	*0*	-	-	
65-74	*0.02*	-	-	
≥75	*0.07*	-	-	
Utility UK general population	*By age*	-	-	Kind et al [35]
All-cause mortality	*By age*	-	-	ONS [31]
Discount rate costs	*3.5%*	-	-	NICE [28]
Discount rate QALYs	*3.5%*	-	-	NICE **[28]**

†The 28-day risk of death in the placebo arm was sampled from a beta distribution during the probabilistic sensitivity analysis, based on the proportion of deaths during the trial period (548/5981). The deaths occurring on each day, as a proportion of the overall 28-day risk, remained the same during sampling.

For peer review only - http://bmjopen.bmj.com/site/about/guidelines.xhtml

Table 2: Base case cost parameters

Parameter	Value	SE or proportion	Distribution	Source
TXA administration	*£30.01*	-	-	BNF [36], PSSRU[29], Assumption
Inpatient stays to day-28				
Total length of stay (days)				
TXA	*5.83*	1.46	Gamma	HALT-IT
No TXA	*5.80*	1.47	Gamma	HALT-IT
Days in ICU				
TXA	*0.4*	0.46	Gamma	HALT-IT
No TXA	*0.4*	0.51	Gamma	HALT-IT
Unit cost per day non-ICU	*£338*	-	-	Reference costs [39]
Unit cost per day on ICU	*£1,594*	-	-	Reference costs [39]
Procedures to day-28				
Prob endoscopy - diagnostic				
TXA	*0.8*	4,781/5,953	Beta	HALT-IT
No TXA	*0.79*	4,729/5,978	Beta	HALT-IT
Unit cost	*£665*	-	-	Reference costs [39]
Prob endoscopy - therapeutic				
TXA	*0.43*	2,542/5,953	Beta	HALT-IT
No TXA	*0.44*	2,658/5,978	Beta	HALT-IT
Unit cost	*£777*	-	-	Reference costs [39]
Prob surgical intervention				
TXA	*0.02*	146/5,953	Beta	HALT-IT
No TXA	*0.03*	158/5,978	Beta	HALT-IT
Unit cost	*£1,377*	-	-	Reference costs [39]
Prob radiological - diagnostic				
TXA	*0.29*	1,704/5,953	Beta	HALT-IT
No TXA	*0.29*	1,744/5,978	Beta	HALT-IT
Unit cost	*£4,986*	-	-	Reference costs [39]
Prob radiological - therapeutic				
TXA	*0.01*	74/5,953	Beta	HALT-IT
No TXA	*0.01*	89/5,978	Beta	HALT-IT
Unit cost	*£4,986*	-	-	Reference costs [39]
Transfusions				
Prob blood or red cells				
TXA	*0.67*	3,984/5,951	Beta	HALT-IT
No TXA	*0.67*	4,018/5,978	Beta	HALT-IT
Mean units of blood or red cells				
TXA	*2.80*	0.61	Gamma	HALT-IT
No TXA	*2.90*	0.69	Gamma	HALT-IT
Unit cost	*£132*	-	-	NHS Blood & trans. [40]
Prob fresh frozen plasma				
TXA	*0.15*	910/5,951	Beta	HALT-IT
No TXA	*0.16*	993/5,978	Beta	HALT-IT
Mean units fresh frozen plasma				
TXA	*0.90*	0.61	Gamma	HALT-IT
No TXA	*1.0*	0.66	Gamma	HALT-IT
Unit cost	*£31*	-	-	NHS Blood & trans. [40]
Prob platelets				
TXA	*0.04*	219/5,951	Beta	HALT-IT
No TXA	*0.04*	255/5,978	Beta	HALT-IT
Mean units of platelets				
TXA	*0.20*	0.23	Gamma	HALT-IT
No TXA	*0.20*	0.26	Gamma	HALT-IT
Unit cost	*£192*	-	-	NHS Blood & trans. [40]
Post discharge costs				
Year1	*£4,350*	£2,175†	Gamma	Ramagopalan et al. [41]
Year 2	*£1,980*	£990†	Gamma	Ramagopalan et al. [41]
Year 3	*£1,938*	£969†	Gamma	Ramagopalan et al. **[41]**

†standard errors were assumed to be 50% of the mean value in the base case analysis

For peer review only

Table 3: Base case deterministic results, including a breakdown of cost components

	No TXA	TXA
Total costs	£18,220	£18,155
TXA costs	£0	£30
Procedures and transfusions	£5,162	£5,112
Monitoring costs	£13,057	£13,013
Life Years	12.08	12.04
QALYs	8.61	8.58
Incremental costs		-£64.30
Incremental QALYs		-0.041
ICER(LY)		£1,576
ICER (QALY)		£2,209

For peer review only

Table 4: Deterministic one-way sensitivity analysis results

	Inc. costs (£)†	Inc. LY†	Inc. QALY†	ICER LY (£)	ICER QALY (£)	INMB‡ (QALY) (£)
**Base case**	-64	-0.041	-0.029	1,576	2,209	-518
Monitoring costs (full monitoring costs for year 4 onwards)	-106	-0.041	-0.029	2,605	3,652	-476
Monitoring costs not included beyond 3 years	-43	-0.041	-0.029	1,061	1,488	-539
0% discount rate	-78	-0.060	-0.043	1,291	1,828	-775
6% discount rate	-58	-0.032	-0.023	1,799	2,508	-406
Utility post discharge (0.5)	-64	-0.041	-0.020	1,576	3,297	-326
Monitoring costs - inpatient costs excluded in year 1	-55	-0.041	-0.029	1,341	1,880	-527
TXA all-cause mortality risk ratio (0.92)	85	0.097	0.069	875*	1,227*	1304
TXA all-cause mortality risk ratio (1.16)	-231	-0.195	-0.139	1,185	1,662	-2548
Total TXA administration at £0	-94	-0.041	-0.029	2,311	3,240	-488

†Negative costs, LYs and QALYs indicate that TXA is less costly and less effective than no TXA.
‡ Incremental net monetary benefit (INMB) calculated using a £20,000 willingness to pay for an additional QALY threshold.
*In this scenario, the ICERs flip meaning they favour TXA treatment (hence the positive INMB).

For peer review only - http://bmjopen.bmj.com/site/about/guidelines.xhtml

Figures

Figure 1: Markov Model structure showing both health states within the model.

For peer review only

For peer review only - http://bmjopen.bmj.com/site/about/guidelines.xhtml

Figure 2: Base case cost-effectiveness plane

The dotted line represents the £20,000 willingness to pay threshold; points falling on or below this line are considered cost-effective.
The NE quadrant contains 15% of simulations; SW quadrant 38%; SE quadrant 14%; NW quadrant 33%

For peer review only

1 *Figure 3: Base case cost-effectiveness acceptability curve*

2

3

For peer review only - http://bmjopen.bmj.com/site/about/guidelines.xhtml

Statements

Contributorship statement: All authors (NB, AM, IR, HSS, VJ and JW) contributed to the study conception and design. The economic model was developed by NB and JW. The economic analyses were performed by NB, AM, and JW. All authors (NB, AM, IR, HSS, VJ and JW) were involved in the interpretation of the study results. The first draft of the manuscript was written by NB. All authors (NB, AM, IR, HSS, VJ and JW) provided critical review of the manuscript, and have read and approved the final manuscript.

Competing interests: The authors declare that they have no financial or non-financial interests to disclose, and no conflicts of interest to declare.

Funding: This work was supported by the UK National Institute for Health Research Health Technology Assessment Programme (HTA/11/01/04).

Data sharing statement: For the HALT-IT trial data, individual de-identified patient data, including data dictionary, will be made available via our data sharing portal, The Free Bank of Injury and Emergency Research Data (freeBIRD) website indefinitely (https://freebird.lshtm.ac.uk/). The economic model is available from the authors upon request.

Ethics approval: The HALT-IT trial was approved by the UK NRES Committee East of England (reference number 12/EE/0038), and by the national and local research ethics committees in all participating non-UK countries.

For peer review only - http://bmjopen.bmj.com/site/about/guidelines.xhtml

References

1. Hearnshaw, S., et al., *Acute upper gastrointestinal bleeding in the UK: Patient characteristics, diagnoses and outcomes in the 2007 UK audit.* Gut, 2011;60(1327-35.
2. McPherson, S., et al., *Gastrointestinal Haemorrhage: Time to Get Control.* London, UK: National Confidential Enquiry into Patient Outcome and Death, 2015;
3. Crooks, C., T. Card, and J. West, *Reductions in 28-day mortality following hospital admission for upper gastrointestinal hemorrhage.* Gastroenterology, 2011;141(1):62-70.
4. Henderson, R. *Oesophageal Varices*. Gastroenterology 2016 [cited 2020 18 July]; Available from: https://patient.info/doctor/oesophageal-varices#.
5. Hwang, J.H., et al., *The role of endoscopy in the management of acute non-variceal upper GI bleeding.* Gastrointestinal Endoscopy, 2012;75(6):1132-1138.
6. Campbell, H.E., et al., *Costs and quality of life associated with acute upper gastrointestinal bleeding in the UK: cohort analysis of patients in a cluster randomised trial.* BMJ open, 2015;5(4):e007230.
7. Wallis, J.P., A.W. Wells, and C.E. Chapman, *Changing indications for red cell transfusion from 2000 to 2004 in the North of England.* Transfusion Medicine, 2006;16(6):411-417.
8. Villanueva, C., et al., *Transfusion Strategies for Acute Upper Gastrointestinal Bleeding.* New England Journal of Medicine, 2013;368(1):11-21.
9. Dunn, C.J. and K.L. Goa, *Tranexamic acid.* Drugs, 1999;57(6):1005-1032.
10. Vermylen, J., et al., *A double blind study of the effect of tranexamic acid in essential menorrhagia.* Thrombosis and Haemostasis, 1968;20(02):583-587.
11. Srinil, S. and U. Jaisamrarn, *Treatment of idiopathic menorrhagia with tranexamic acid.* JOURNAL-MEDICAL ASSOCIATION OF THAILAND, 2005;88(S1.
12. Wardrop, D., et al., *Antifibrinolytics (lysine analogues) for the prevention of bleeding in patients with haematological disorders.* Cochrane Database of Systematic Reviews, 2013;7):
13. Watterson, C. and N. Beacher, *Preventing perioperative bleeding in patients with inherited bleeding disorders.* Evidence-Based Dentistry, 2017;18(1):28-29.
14. Nutbeam, T., *In adult patients presenting as emergencies with upper gastrointestinal bleeding, does tranexamic acid decrease mortality?* African Journal of Emergency Medicine, 2015;5(2):85-92.
15. The CRASH-3 Trial Collaborators, *Effects of tranexamic acid on death, disability, vascular occlusive events and other morbidities in patients with acute traumatic brain injury (CRASH-3): a randomised, placebo-controlled trial.* The Lancet, 2019;394(10210):1713-1723.
16. Shakur, H., et al., *Effect of early tranexamic acid administration on mortality, hysterectomy, and other morbidities in women with post-partum haemorrhage (WOMAN): an international, randomised, double-blind, placebo-controlled trial.* The Lancet, 2017;389(10084):2105-2116.
17. Olldashi, F., et al., *Effects of tranexamic acid on death, vascular occlusive events, and blood transfusion in trauma patients with significant haemorrhage (CRASH-2): a randomised, placebo-controlled trial.* The Lancet, 2010;376(9734):23-32.
18. Roberts, I., D. Prieto-Merino, and D. Manno, *Mechanism of action of tranexamic acid in bleeding trauma patients: an exploratory analysis of data from the CRASH-2 trial.* Critical Care, 2014;18(6):685.
19. Roberts, I., et al., *Effects of a high-dose 24-h infusion of tranexamic acid on death and thromboembolic events in patients with acute gastrointestinal bleeding (HALT-IT): an international randomised, double-blind, placebo-controlled trial.* The Lancet, 2020;395(10241):1927-1936.
20. Hawkey, G., et al., *Drug treatments in upper gastrointestinal bleeding: value of endoscopic findings as surrogate end points.* Gut, 2001;49(3):372-379.
21. Cormack, F., et al., *Tranexamic acid in upper gastrointestinal haemorrhage.* The Lancet, 1973;301(7814):1207-1208.
22. Bennett, C., et al., *Tranexamic acid for upper gastrointestinal bleeding.* Cochrane Database of Systematic Reviews, 2014;11):
23. Twum-Barimah, E., et al., *Systematic review with meta-analysis: the efficacy of tranexamic acid in upper gastrointestinal bleeding.* Alimentary Pharmacology & Therapeutics, 2020;
24. National Institute for Health and Care Excellence, *Acute upper gastrointestinal bleeding in over 16s: management [CG141].* 2012;
25. Oakland, K., et al., *Diagnosis and management of acute lower gastrointestinal bleeding: guidelines from the British Society of Gastroenterology.* Gut, 2019;68(5):776.
26. Roberts, I., et al., *HALT-IT - tranexamic acid for the treatment of gastrointestinal bleeding: study protocol for a randomised controlled trial.* Trials, 2014;15(1):450.

For peer review only - http://bmjopen.bmj.com/site/about/guidelines.xhtml

27. Roberts, I., et al., *A high-dose 24-hour tranexamic acid infusion for the treatment of significant gastrointestinal bleeding: HALT-IT RCT.* Health Technol Assess, 2021;25(58):1-86.
28. National Institute for Health and Care Excellence, *Guide to the methods of technology appraisal.* National Institute for Health and Clinical Excellence (NICE) London, UK, 2018;
29. Jones, K. and A. Burns, *Unit Costs of Health and Social Care 2021*, in *Personal Social Services Research Unit*. 2021, University of Kent: Canterbury.
30. Williams, J., et al., *Cost-effectiveness analysis of tranexamic acid for the treatment of traumatic brain injury, based on the results of the CRASH-3 randomised trial: a decision modelling approach.* BMJ global health, 2020;5(9):
31. Office for National Statistics. *National life tables, UK: 2016 to 2018*. Available from: https://www.ons.gov.uk/peoplepopulationandcommunity/birthsdeathsandmarriages/lifeexpectancies/bulletins/nationallifetablesunitedkingdom/2016to2018.
32. Crooks, C.J., T.R. Card, and J. West, *Excess long-term mortality following non-variceal upper gastrointestinal bleeding: a population-based cohort study.* PLoS medicine, 2013;10(4):e1001437.
33. Jairath, V., et al., *Restrictive versus liberal blood transfusion for acute upper gastrointestinal bleeding (TRIGGER): a pragmatic, open-label, cluster randomised feasibility trial.* Lancet, 2015;386(9989):137-44.
34. Katz, S., et al., *Studies of the illness in the aged, the index of ADL: A standarsized measure of biological and psychological functioning.* JAMA, 1963;185(914-9.
35. Kind, P., et al., *Variations in population health status: results from a United Kingdom national questionnaire survey.* BMJ, 1998;316(
36. Royal Pharmaceutical Society. *British National Formulary*. 2019 14/02/2022]; Available from: https://about.medicinescomplete.com/publication/british-national-formulary/.
37. Guerriero, C., et al., *Cost-Effectiveness Analysis of Administering Tranexamic Acid to Bleeding Trauma Patients Using Evidence from the CRASH-2 Trial.* PLoS One, 2011;6(5):e18987.
38. Li, B., et al., *Tranexamic acid for treatment of women with post-partum haemorrhage in Nigeria and Pakistan: a cost-effectiveness analysis of data from the WOMAN trial.* The Lancet. Global health, 2018;6(2):e222-e228.
39. NHS Improvement. *National schedule of reference costs 2017/18*. Available from: https://webarchive.nationalarchives.gov.uk/20200501111106/https://improvement.nhs.uk/resources/reference-costs/.
40. NHS Blood and Transport. *NHS Blood and Transport Price List 2018/2019*. Available from: https://nhsbtdbe.blob.core.windows.net/umbraco-assets-corp/14810/price_list_bc_nhs_2018-19.pdf.
41. Ramagopalan, S.V., et al., *Costs of gastrointestinal bleeding events in atrial fibrillation: a UK Clinical Practice Research Datalink study.* Future cardiology, 2019;15(5):367-375.
42. Gayet-Ageron, A., et al., *Effect of treatment delay on the effectiveness and safety of antifibrinolytics in acute severe haemorrhage: a meta-analysis of individual patient-level data from 40 138 bleeding patients.* The Lancet, 2018;391(10116):125-132.

For peer review only

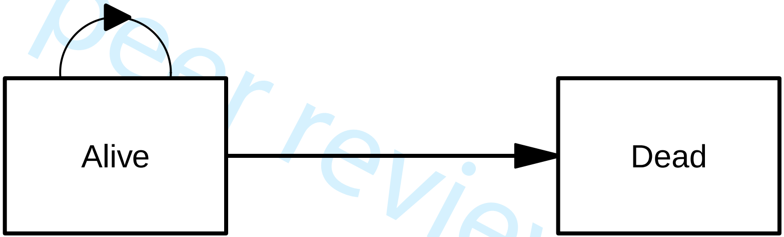

For peer review only

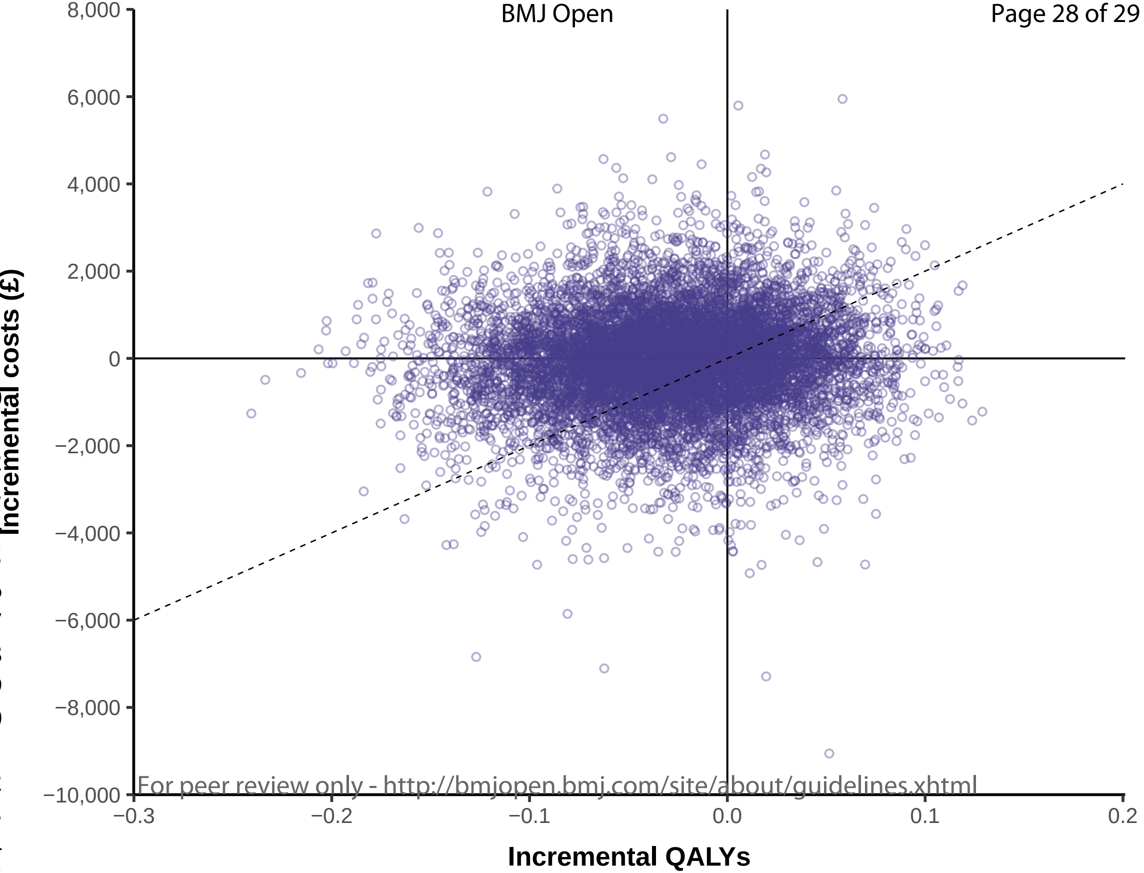

For peer review only - http://bmjopen.bmj.com/site/about/guidelines.xhtml

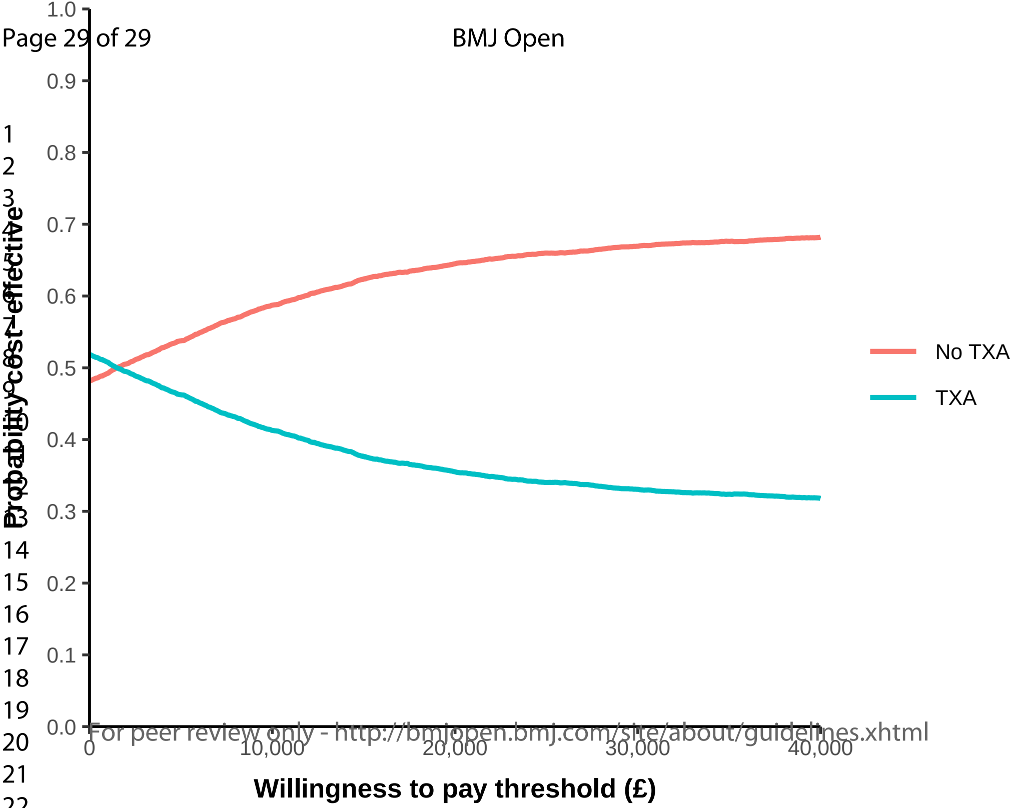
1.0
0.9
0.8
0.7
0.6
0.5
0.4
0.3
0.2
0.1
0.0
Probability cost−effective
0
10,000
20,000
30,000
40,000
Willingness to pay threshold (£)
No TXA
TXA

Table 1 | The CHEERS 2022 checklist

Section/topic	Item No	Guidance for reporting	Reported in section
**Title**			
Title	1	Identify the study as an economic evaluation and specify the interventions being compared.	Front Page
**Abstract**			
Abstract	2	Provide a structured summary that highlights context, key methods, results, and alternative analyses.	Abstact
**Introduction**			
Background and objectives	3	Give the context for the study, the study question, and its practical relevance for decision making in policy or practice.	Introduction
**Methods**			
Health economic analysis plan	4	Indicate whether a health economic analysis plan was developed and where available.	Introduction
Study population	5	Describe characteristics of the study population (such as age range, demographics, socioeconomic, or clinical characteristics).	Analysis
Setting and location	6	Provide relevant contextual information that may influence findings.	Analysis
Comparators	7	Describe the interventions or strategies being compared and why chosen.	Analysis
Perspective	8	State the perspective(s) adopted by the study and why chosen.	Analysis
Time horizon	9	State the time horizon for the study and why appropriate.	Analysis
Discount rate	10	Report the discount rate(s) and reason chosen.	Analysis
Selection of outcomes	11	Describe what outcomes were used as the measure(s) of benefit(s) and harm(s).	Analysis / Utilities
Measurement of outcomes	12	Describe how outcomes used to capture benefit(s) and harm(s) were measured.	Analysis / Treatment Effect / Utilities
Valuation of outcomes	13	Describe the population and methods used to measure and value outcomes.	Utilities
Measurement and valuation of resources and costs	14	Describe how costs were valued.	Costs
Currency, price date, and conversion	15	Report the dates of the estimated resource quantities and unit costs, plus the currency and year of conversion.	Analysis
Rationale and description of model	16	If modelling is used, describe in detail and why used. Report if the model is publicly available and where it can be accessed.	Model Structure
Analytics and assumptions	17	Describe any methods for analysing or statistically transforming data, any extrapolation methods, and approaches for validating any model used.	Model Structure / Treatment Effect
Characterising heterogeneity	18	Describe any methods used for estimating how the results of the study vary for subgroups.	Analysis
Characterising distributional effects	19	Describe how impacts are distributed across different individuals or adjustments made to reflect priority populations.	Analysis
Characterising uncertainty	20	Describe methods to characterise any sources of uncertainty in the analysis.	Sensitivity Analysis
Approach to engagement with patients and others affected by the study	21	Describe any approaches to engage patients or service recipients, the general public, communities, or stakeholders (such as clinicians or payers) in the design of the study.	Public and Patient Involvement
**Results**			
Study parameters	22	Report all analytic inputs (such as values, ranges, references) including uncertainty or distributional assumptions.	Table 1-2 (and Methods)
Summary of main results	23	Report the mean values for the main categories of costs and outcomes of interest and summarise them in the most appropriate overall measure.	Table 3 / Base Case Analysis
Effect of uncertainty	24	Describe how uncertainty about analytic judgments, inputs, or projections affect findings. Report the effect of choice of discount rate and time horizon, if applicable.	PSA / DSA / Meta-Analysis Scenario (all in Results section)
Effect of engagement with patients and others affected by the study	25	Report on any difference patient/service recipient, general public, community, or stakeholder involvement made to the approach or findings of the study	N/A - Engagement infuenced trial design
**Discussion**			
Study findings, limitations, generalisability, and current knowledge	26	Report key findings, limitations, ethical or equity considerations not captured, and how these could affect patients, policy, or practice.	Discussion
**Other relevant information**			
Source of funding	27	Describe how the study was funded and any role of the funder in the identification, design, conduct, and reporting of the analysis	Statements
Conflicts of interest	28	Report authors conflicts of interest according to journal or International Committee of Medical Journal Editors requirements.	Statements

available health economic evaluations, as well as the availability of CHEERS in multiple languages since 2013, there is some indication CHEERS could be more widely and appropriately used. A convenience sample of 50 articles citing CHEERS revealed only 42% (95% confidence interval 28% to 56%) made an appropriate use of CHEERS.[5] This is a similar rate to those observed with other major reporting guidelines (CONSORT, PRISMA, ARRIVE). The same study also found that the inappropriate use of CHEERS has increased from its time of publication.

In creating this update, we also wanted to ensure the broadest possible application of CHEERS. Previous concerns raised about its lack of applicability in cost-benefit analyses (CBAs) were understandable, given original CHEERS guidance leaning strongly towards proving direction for those conducting cost-effectiveness analyses (including cost-utility analyses). This was driven, in part, by the small prevalence and impact of published CBAs at the time of the original CHEERS guidance. However, it is clear that broader characterisations of the benefits of healthcare, in concert with the promotion and publication of other forms of economic evaluation, such as distributional cost-effectiveness analysis, are becoming increasingly important. Health economic evaluation is also finding increasing application across a wider spectrum of health interventions. We hope the revised CHEERS statement addresses these concerns.

For peer review only - http://bmjopen.bmj.com/site/about/guidelines.xhtml

BMJ: first published as 10.1136/bmj-2021-067975 on 11 January 2022. Downloaded from http://www.bmj.com/ on 1 April 2022 by guest. Protected by copyright.
